# Solvent‐Dependent Reactivity and Photochemistry of Dinuclear and Mononuclear Platinum(IV) Azido Triazaolato Complexes

**DOI:** 10.1002/ejic.202100041

**Published:** 2021-03-16

**Authors:** Kezi Yao, Arnau Bertran, Jacques Morgan, Charlotte Greenhalgh, Katharina Edkins, Alice M. Bowen, Nicola J. Farrer

**Affiliations:** ^1^ Chemistry Research Laboratory University of Oxford 12 Mansfield Road Oxford OX1 3TA United Kingdom; ^2^ Centre for Advanced Electron Spin Resonance and Inorganic Chemistry Laboratory, Department of Chemistry University of Oxford South Parks Road Oxford OX1 3QR United Kingdom; ^3^ School of Health Sciences University of Manchester Oxford Road Manchester M13 9PL United Kingdom; ^4^ The Department of Chemistry, Photon Science Institute and the National EPR Research Facility University of Manchester Oxford Road Manchester M13 9PL United Kingdom

**Keywords:** Azides, Photoactivity, Platinum, Radicals, Structure elucidation

## Abstract

Reaction between the platinum(IV) azido complex *trans,trans*,*trans*‐[Pt(py)_2_(N_3_)_2_(OH)_2_] (**1**) and 1,4‐diphenyl‐2‐butyne‐1,4‐dione **2** in MeCN produces the intermediate peroxide‐bridged dimeric platinum(IV) azido triazolato species (**5**), which has been characterised by X‐ray crystallography. However, if the reaction between **1** and **2** is conducted in MeOH it results in decomposition. Over time in MeCN, dimer (**5**) converts into mononuclear complexes *trans,trans*,*trans*‐[Pt(py)_2_(N_3_)(triazole)(OH)_2_] (**3 a**/**3 b**), which are in dynamic exchange. If resuspended in protic solvents (MeOH,H_2_O), **3 a**/**3 b** undergo a slow (22 d) irreversible rearrangement to a cyclised platinum(IV) species **4** which contains a formally N,O‐chelated ligand. Conversion of **3 a**/**3 b** to **4** in *d*_4_‐MeOH can be accelerated (384x) by irradiation with visible light, although continued irradiation also produces N_3_^.^ and OH^.^ radicals, and the [**4**‐N_3_]^+^ species can be readily detected by ESI‐MS. Solvent choice significantly effects both the cycloaddition reaction between **1** and **2**, and the stability of the resultant complexes.

## Graphical abstract

The choice of solvent is crucial to the success of cycloaddition reactions with platinum(IV) azido complexes. Following synthesis in acetonitrile, rearrangement of the triazole can occur in protic solvents. Irradiation with visible light induces azido radical release, without reduction to Pt^II^ species, due to cyclometallation of the complex.

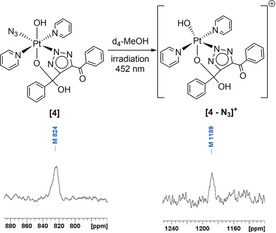

## Introduction

1,3‐dipolar cycloaddition reactions of metal azido complexes with acetylenes to form 1,2,3‐triazoles are a well‐established route to novel metal complexes.^[1]^ These reactions have been previously explored by other groups for platinum(II) complexes[^2^, ^3^, ^4^, ^5^] and by us for platinum(IV) azido complexes.[^6^, ^7^, ^8^] Inorganic platinum‐click reactions enable the synthesis of novel (potentially multimetallic) platinum complexes and metallopolymers.^[9]^ They can also provide plausible routes to introduce functional groups for fluorescent monitoring^[4]^ and to monitor Pt‐azido drug localisation.^[10]^ The cycloaddition reaction results in the formation of 1,2,3‐triazoles, and these organic ligands can show promising anti‐cancer activity both in their own right^[11]^ and as components of metal complexes.^[12]^ Triazoles are also widely employed as chemosensors.^[13]^ Understanding the solution chemistry and photochemistry of metal triazole complexes is crucially important for further rational design.

**Scheme 1:**
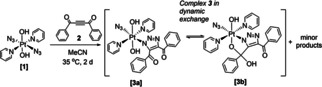
Reaction of *trans,trans,trans*‐[Pt(N_3_)_2_(OH)_2_(py)_2_] (**1**) with 1,4‐diphenyl‐2‐butyne‐1,4‐dione (**2**) showing formation of **3** (**3 a**/**3 b**) as well as other minor products.

**Scheme 2:**
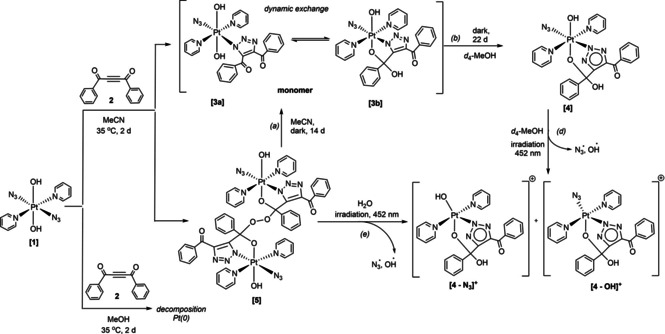
Reaction of *trans,trans,trans*‐[Pt(N_3_)_2_(OH)_2_(py)_2_] (**1**) with 1,4‐diphenyl‐2‐butyne‐1,4‐dione (**2**), showing pathways of reactivity to different products and the effect of different solvents. The presence of H_2_O_2_ enables greater production of **5**, and results in low‐level production of OH radicals in the dark during transofrmation (a), in accordance with reports for other platinum(IV) complexes.^[15]^

We previously reported that the platinum(IV) azido complex **1** undergoes a spontaneous reaction with acetylene **2** in MeCN to produce complex **3**, a *mono* triazolato cycloaddition product, which exists as an equilibrium between two rapidly interconverting species; **3 a** and **3 b** (Scheme 1).^[7]^ A small amount of another complex – **5** – was also detected during HPLC purification of **3 a**/**3 b** but we previously were unable to isolate sufficient quantities of **5** for further characterisation. We now report that compound **5** is a peroxide‐bridged dimer, which slowly evolves over time in the dark in MeCN to form **3 a**/**3 b** (Scheme 2). We have also determined that both **5** and **3 a**/**3 b** respond to irradiation with blue light. Furthermore, that in protic solvents **3 a**/**3 b** (which exist in equilibrium) evolve into a single cyclised Pt^IV^ complex, **4** which is then stable to further rearrangement. Here we report our findings in detail.

## Synthesis

**Figure 1:**
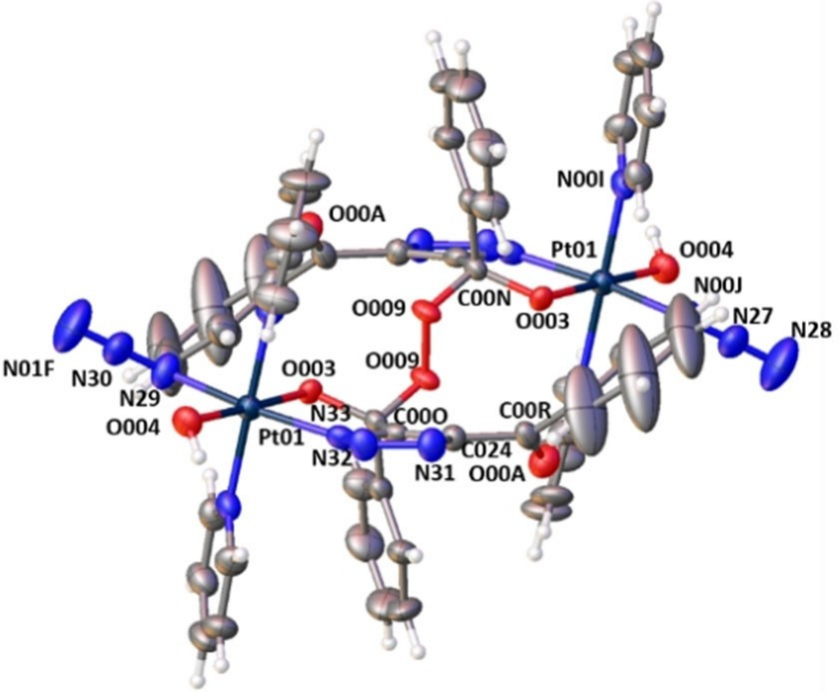
Single crystal X‐ray structure of the peroxide dimer **5** with thermal ellipsoids displayed at 50 % probability.^[16]^ For clarity, only key atoms discussed in the text are labelled here, the fully labelled structure is given in Figure S4. Colour code: carbon (grey), nitrogen (blue), oxygen (red), hydrogen (light grey), platinum (cyan).

*Trans, trans, trans*‐[Pt(N_3_)_2_(OH)_2_(py)_2_] (**1**) was synthesised as previously reported, the final step of the synthesis involving oxidation with H_2_O_2_ followed by crystallisation.^[14]^ We previously reported that when the reaction was conducted in MeCN, both the main product **3 a**/**3 b** ^[7]^ and several minor species including **5** were successfully obtained, without any observed decomposition. However, here we report that when the cycloaddition reaction between **1** and 1,4‐diphenyl‐2‐butyne‐1,4‐dione (**2**) is conducted in MeOH (35 °C, 2 d), it results in decomposition, with formation of a black platinum(0) precipitate (Scheme 2). The solvent choice for the cycloaddition therefore has a significant effect on the reaction. Complex **5** (*t*_R_=4.01 min) was previously challenging to isolate, since it formed as a minor product in the reaction (7 %), eluting immediately before **3 a**/**3 b** (*t*_R_=4.31 min) during HPLC purification (Figure S1). However, we now report that if **1** is not purified by HPLC or recrystallised before use (therefore retaining residual H_2_O_2_ from the final synthetic step) the quantity of **5** observed significantly increases, such that it forms 31 % of the total integrated HPLC trace (UV‐Vis detection, Figure S2), making it considerably easier to isolate as the 5 ⋅ H_2_O_2_ adduct, and to fully characterise (Figure S3). Historically, platinum(IV) complexes such as *cis,cis,trans*‐[Pt^IV^Cl_2_(NH_3_)_2_(OH)_2_] have shown a propensity for co‐crystallisation of H_2_O_2_.^[15]^ We were initially unaware that **5** was a dinuclear species, since the molecular weight of the compound exceeded the detection limit of the HPLC mass spectrometer (1250 *m/z*), and the ESI‐MS conditions did not favour the detection of multiply charged ions. Therefore, 5 ⋅ H_2_O_2_ was observed by HPLC via both UV‐Vis absorption and the detection of the common fragmentation product [3‐OH]^+^ at 688.05 *m/z* (Figure S2, *bottom*). This rendered it indistinguishable from complex **3 a**/**3 b** which results in the same fragmentation species in the HPLC. Following HPLC purification, crystals of **5** were grown from MeCN, and were shown by X‐ray crystallography to consist of a peroxide‐bridged dimer, with no H_2_O_2_ observed in the crystal lattice (Figure 1).

The X‐ray crystallographic structure of **5** has an O−O bond length of 1.475(8) Å, consistent with a bond order of **1** ^[17]^ and it also exhibits a C00N−O009(peroxide) bond length of 1.432(8) Å. The Pt−N azido (Pt−N29−N30−N01F) ligand shows slightly greater linearity (176.8(9)°) than is observed in the azido ligand(s) contained in both **3 b** and **1**. The bond lengths within the azido ligand are very similar to **3 b** and to **1**; 1.210(9) Å (N29−N30) and 1.147(9) Å (N30−N01F). The angle subtended at Pt−N29−N30 (116.0(5)°) is intermediate between that seen for complex **3 b** and complex **1**, and the Pt−N33 triazole bond length is 2.011(6) Å, slightly elongated in comparison to **3 b**.^[7]^

IR spectroscopy of a MeOH solution of **5** ⋅ H_2_O_2_ showed a strong ν_asym_N_3_ stretch at 2049.04 cm^−1^ (Figure S5), consistent with the presence of the azido groups.

If the cycloaddition was carried out with **1** which had residual H_2_O_2_ remaining from the syntheses, **5** was recovered from the HPLC and observed by ESI‐MS as the H_2_O_2_ adduct; [**5** ⋅ H_2_O_2_+Na]^+^ at 1465.2604 *m/z* (confirmed by HRMS, error 5.59 ppm, Figure S6). Collision‐induced dissociation studies (MS/MS) of [**5 a** ⋅ H_2_O_2_+Na]^+^ at 1465.00 *m/z* revealed facile fragmentation by homolytic cleavage of the peroxide bond and retention of the sodium to give [Pt(OH)_2_(N_3_)(triazole)(py)_2_O+Na]^+^ as a stable species at 744.22 *m/z* as well as other minor species (Figure S7). The species at 744.03 *m/z* observed in the low‐resolution ESI‐MS of **5** (Figure S1) could therefore correspond to this same fragmentation product.

^195^Pt NMR spectroscopy of **5** ⋅ H_2_O_2_ (in *d*_3_‐MeCN) showed a single resonance at 776 ppm (Figure S8). In the same solvent, the monomers **3 a** (689 ppm) and **3 b** (785 ppm) give rise to two resonances.^[7]^ This suggests that dynamic exchange processes similar to those seen for **3 a**/**3 b** do not occur in **5**, consistent with the carbonyl/hydroxyl group of the triazole ligand being part of the stable peroxide bond.

The ^1^H NMR spectrum could be assigned in agreement with the proposed structure of **5**, although determination of the py‘ and py rings are tentative (Figures S9, S10, S11). A singlet was observed for **5** ⋅ H_2_O_2_ at 9.16 ppm, which underwent exchange with the residual water peak (^1^H NMR NOESY, Figure S12); this may correspond to protons of the associated/coordinated H_2_O_2_. Assignment of the ^13^C NMR spectra (Figure S13) was aided by HSQC and HMBC experiments (Figures S14 and S15 respectively). Many features of the ^13^C NMR spectrum of **5** ⋅ H_2_O_2_ were similar to that of **3 b**, including the observation of a ^2^ *J*_CPt_ coupling of 43.8 Hz for one of the quaternary triazole carbon resonances in **5** ⋅ H_2_O_2_ (151.5 ppm). The quaternary carbon connected to the Pt−O group, the Ph ring and the peroxide bridge was observed at 112.1 ppm (showing a 3‐bond HMBC correlation to the Ph_ortho_ protons). This was at higher field than for complex **3 b**, in which the sp^3^ carbon coordinated to an OH group rather than a O−O group was observed at 103.0 ppm. The UV‐Vis absorbance spectrum of **5** ⋅ H_2_O_2_ was broadly similar to the spectrum of **3 a**/**3 b** (Figure S16).

## Conversion of 5 ⋅ H_2_O_2_ to 3 a/3 b in *d*_3_‐MeCN

Both **5** ⋅ H_2_O_2_ and **3 a**/**3 b** were isolated as pale yellow powders, although **5** ⋅ H_2_O_2_ was noticeably less soluble than **3 a**/**3 b** in organic solvents (MeCN, MeOH). Complex **5** ⋅ H_2_O_2_ was unstable in MeCN solution, changing over time in the absence of light. Comparing ^1^H NMR spectra obtained immediately following dissolution (Figure S9) with 2D ^1^H and ^13^C NMR spectra acquired later the same day, new resonances can already be seen appearing in the later spectra (for example * in Figure S10).

Monitoring this conversion by cw‐EPR in the presence of the spin trap DMPO (Figure S17) revealed a build‐up of a small amount of a nitroxide species – over several weeks – in MeCN (Figure S18), showing hyperfine coupling only to a single nitrogen. No radical species were detected in the analogous spin trapping experiment performed with **3 a**/**3 b** in MeCN (Figure S19). When the same experiments were carried out in water, a small amount of a DMPO^.^−OH radical adduct was observed over several days with **5** ⋅ H_2_O_2_, indicating the slow formation and subsequent trapping of OH^.^ (Figure S20), while no radical species were detected under the same conditions from **3 a**/**3 b** in H_2_O (Figure S21). We suggest this is due to release of peroxide, which appears to be strongly associated with **5**. Evolution of OH^.^ from platinum(IV) prodrugs containing trace amounts of H_2_O_2_ has been previously reported.^[15]^ Observation of the evolution of OH^.^ in the dark is also compatible with the slow conversion of **5** to **3 a**/**3 b** taking place through homolytic cleavage of the peroxide bridge. In H_2_O, the resulting [**3 b**‐H]^.^ species would produce **3 b** by hydrogen abstraction from the solvent, producing OH^.^, which would be trapped by DMPO. In MeCN, more complicated radical processes could occur, involving degradation of the spin trap by either H_β_ abstraction or cleavage of the N_nitroxidic_−C_α_ bond.^[18]^

## Conversion of 3 a/3 b to 4 in *d*_4_‐MeOH

**Figure 2:**
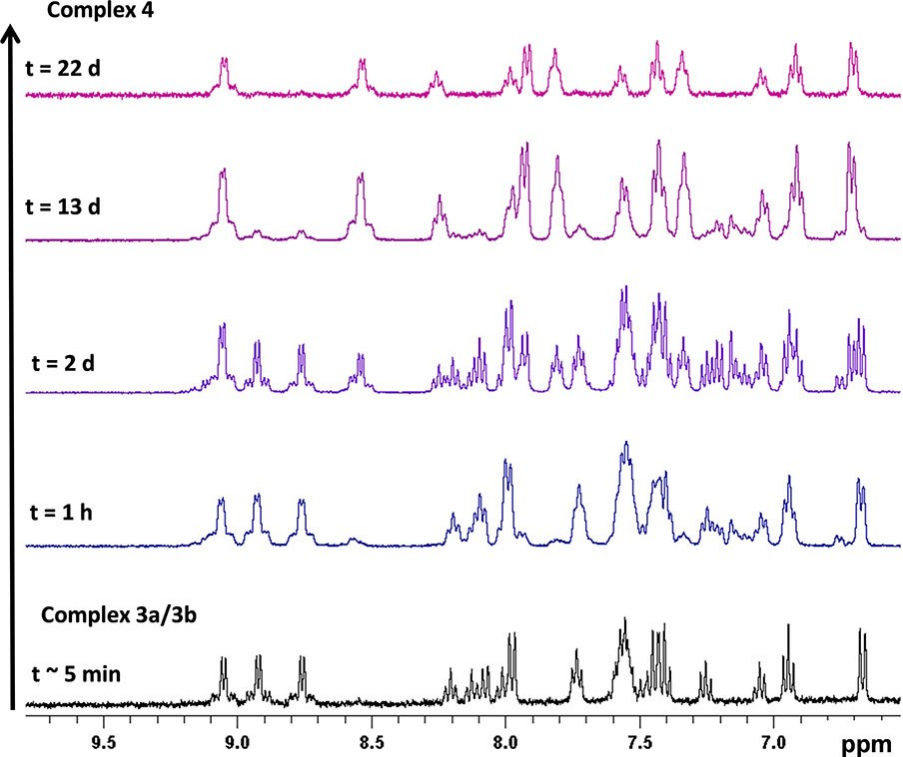
^1^H NMR spectra of **3 a**/**3 b** converting to **4** over 22 d (*d*_4_‐MeOH) at 20 °C.

When the synthesis of **3 a**/**3 b** was conducted in MeCN followed by solvent removal and resuspension of solid **3 a**/**3 b** in protic solvents, it did not result in decomposition. Instead, **3 a**/**3 b** underwent a conversion to a new complex (**4**) in *d*_4_‐MeOH (Scheme 1, Figure 2). By the time ^13^C, ^1^H‐^13^C HSQC and HMBC NMR spectral data had been acquired for a freshly prepared solution of **3 a**/**3 b** in *d*_4_‐MeOH, partial conversion to **4** had already begun. Complete conversion of **3 a**/**3 b** to **4** was relatively slow, with 100 % conversion after 22 d (^1^H NMR spectroscopy, *d*_4_‐MeOH, Figure 2 and Figure S22). During this time, the ^195^Pt NMR resonances at 832 ppm (**3 a**) and 784 ppm (**3 b**) converged to a single resonance at 824 ppm for **4** (*d*_4_‐MeOH). EPR experiments revealed no detectable radical production during the conversion of **3 a**/**3 b** to **4** in the dark (Figure S21).

## Analysis of complex 4

**Figure 3:**
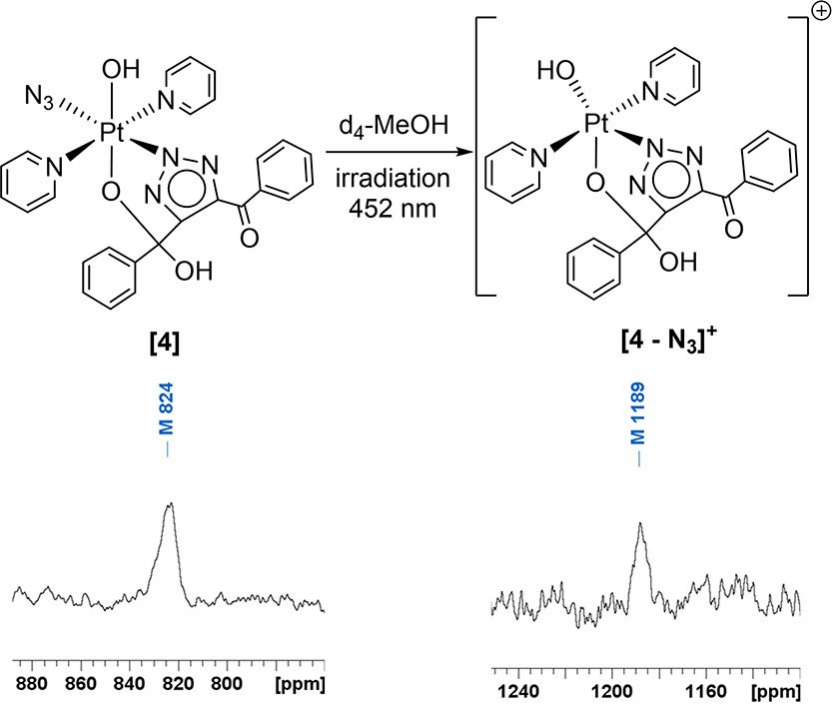
^195^Pt NMR spectra (*d*_4_‐MeOH) of complex **4** (a) before irradiation (86.1 MHz) and (b) after irradiation with blue light, forming [**4**‐N_3_]^+^ (107.6 MHz).

Following completion of the conversion of **3 a**/**3 b** to **4** in *d*_4_‐MeOH, complex **4** (Figure 3) was isolated as a pale yellow solid by rotary evaporation of the solvent, and was fully characterised by ESI‐MS, ^1^H, ^13^C, ^195^Pt and ^14^N NMR spectroscopy, IR and UV‐Vis spectroscopy.

Complex **3 a**/**3 b** had been previously detected by ESI‐MS predominantly as the [**3 a**/**3 b**‐OH]^+^ (688.14 *m/z*) species.^[7]^ Conversion of **3 a**/**3 b** to **4** resulted in similar low‐resolution ESI‐MS: [**4**‐OH]^+^ was detected as the dominant species at 687.89 *m/z*, (C_26_H_21_N_8_O_3_Pt, calcd. 688.13 *m/z*) with [**4**+H]^+^ observed at a significantly lower abundance, at 705.71 *m/z* (C_26_H_23_N_8_O_4_Pt, calcd 706.15 *m/z*). The ESI‐MS were identical regardless of whether the conversion to **4** was carried out in MeOH or *d*_4_‐MeOH, effectively ruling out the possibility that the solvent had been incorporated into the observed species as part of the conversion, since a difference would have been detected due to the difference in molecular weight between the deuterated and undeuterated solvents (Figure S23).

In the ^1^H NMR spectrum of complex **4** in *d*_4_‐MeOH (Figure S24) the resonances in **4** corresponding to Py_para_ and Py_meta_ protons were observed at higher chemical shifts than for **5**, whilst the other aromatic ^1^H NMR resonances were very similar for the two complexes. Complex **4** has two inequivalent Ph rings and two inequivalent Py rings, and NOESY correlations were observed between one pyridyl group and one phenyl ring (Figure S25), in agreement with the proposed structure. Following conversion from **3 a**/**3 b**, complex **4** was stable in solution (*d*_4_‐MeOH) for over 12 months, and speciation remained essentially unchanged (judged by ^1^H NMR spectroscopy and ESI‐MS).

The rearrangement of **3 a**/**3 b** to **4** in MeOH followed by reconstitution of **4** in *d*_3_‐MeCN enabled a direct comparison of **3 a**/**3 b** and **4** in the same solvent (Figure S26). This revealed changes predominantly in the Py_para_ (shielded) Ph_ortho_ (deshielded) and Py_meta_ (shielded) protons but revealed little other structural information. ^13^C NMR spectra were assigned through comparison with previous structures and through ^1^H COSY, ^1^H‐^13^C HSQC, ^1^H‐^13^C HMBC and ^1^H NOESY experiments (Figures S23–S29). The ^14^N NMR spectrum of **4** showed resonances corresponding to the N_β_ (227.0 ppm) and N_γ_/N_triazole_/N_py_ environments (166.2 ppm) (Figure S31). The Pt‐coordinated N_α_ of the azide was anticipated to come into resonance at *ca*. 57 ppm but was broadened beyond detection. As previously observed^[19]^ and subsequently explained by DFT calculations,^[20]^ the quadrupolar interaction of the ^14^N nucleus with the local electric field gradient (EFG) results in significant anisotropic broadening of spectral lines for less symmetric environments, such as the azido N_α_ resonance_._ Since the ^14^N NMR spectrum of **4** provided only limited information we felt it would be unlikely that acquisition of ^14^N NMR of **3** or **5** would provide any additional or useful comparative information. Comparison of IR (*d*_4_‐MeOH) of **3 a**/**3 b** (sample made immediately prior to measurement) and of complex **4** revealed only minor changes; the ν_asym_N_3_ IR stretch being observed at 2048 cm^−1^ for **4** (Figure S32). The UV‐vis spectrum of **4** (Figure S33) was also consistent with **4** being a monoazido complex.

Despite repeated and ongoing attempts, it was not possible to grow crystals of **4** suitable for analysis by X‐ray diffraction. However, the structure proposed for **4** (Figure 3) is consistent with the reported analytical data. We suggest that **4** forms from **3 a**/**3 b** in protic solvents as a result of N1−N2 rearrangement of the triazole ring.^[4]^ An alternative structure for **4** could result from isomerisation at the platinum(IV) centre through exchange of azide and OH (resulting in *cis* OH groups); such isomerisation has been reported for other platinum(IV) complexes, however we suggest that triazole rearrangement is more likely.^[21]^

## Photochemistry of 3 a/3 b, 4 and 5

The slow conversion of **3 a**/**3 b** to **4** in *d*_4_‐MeOH could be accelerated by heat, and was also significantly accelerated (by 384x) through irradiation with light (452 nm) with 80 % conversion observed in 60 min. However, small amounts (<5 %) of [**3**‐N_3_]^+^ (663.1318 *m/z*) were also observed by HPLC after 60 min irradiation, in contrast to conversion in the dark. We chose the irradiation wavelength of 452 nm (blue light) since it is the longest wavelength – of a commercially available LED light source – which affords photochemical conversion of **3 a**/**3 b** within a reasonable length of time. Since the tissue penetration of light is wavelength‐dependent, it is desirable to use the longest wavelength possible to activate these compounds – towards the phototherapeutic window of 630 nm–900 nm. We do however note that successful treatment of superficial tumours has been achieved with blue light, which has the advantage of minimising damage to underlying tissue.^[22]^ We have previously demonstrated using TD‐DFT that platinum(IV) azido complexes have strongly dissociative states at longer wavelengths, which explains why they can be successfully photoactivated with visible light, despite demonstrating only modest absorption at these longer wavelengths.^[14]^

We previously showed that if irradiation of **3 a**/**3 b** is carried out in phosphate buffered saline (PBS) in the presence of 5′‐GMP, no platination of 5′‐GMP was observed.^[7]^ ^195^Pt NMR spectroscopy of **4** (*d*_4_‐MeOH) under irradiation also showed conversion from the resonance at 824 ppm (**4**) to 1189 ppm [**4**‐N_3_]^+^ in the platinum(IV) region of the spectrum (Figure 3). This is consistent with our previous report of the photochemistry of **3 a**/**3 b** (*d*_3_‐MeCN),^[7]^ which showed the ^195^Pt resonances move to a more deshielded position following irradiation and release of azide.

**Figure 4:**
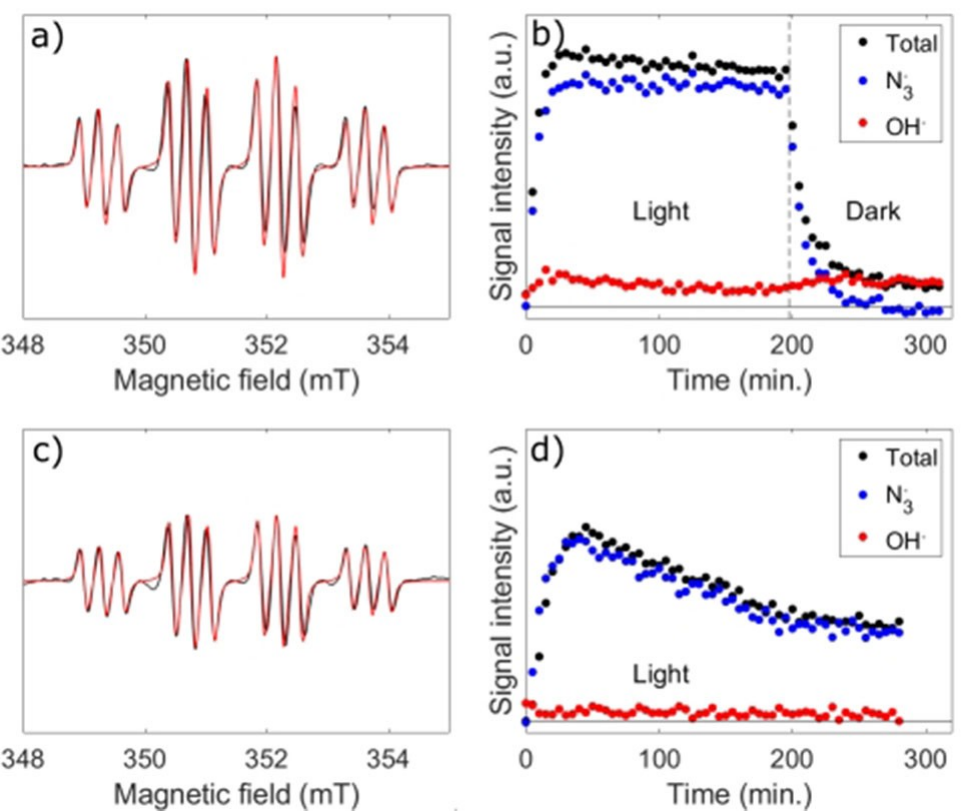
EPR spin trapping experiments under irradiation (440–480 nm) of compounds **3 a**/**3 b** at 10.5 mM in H_2_O (a, b) and 5 ⋅ H_2_O_2_ at <1 mM (c, d) in the presence of 21 mM DMPO. (a, c) Experimental spectra averaged over 100 min of continuous illumination after reaching the maximum signal intensity (black) and simulations with a DMPO^.^−N_3_: DMPO^.^−OH molar ratio of 90 : 10 (a) and 95 : 5 (c) (red). The average total radical adduct concentrations over the first 100 minutes determined from spectral integration are (5.5±0.6) μM (a) and (3.1±0.3) μM (c). (b, d) EPR peak height transients of the total radical adduct signal (black), DMPO^.^−N_3_ signal (blue) and DMPO^.^−OH signal (red). Vertical axes have the same scale in (a, c) and (b, d), respectively, for comparison.

EPR spin‐trapping experiments under irradiation (440–480 nm) were also carried out with freshly prepared **3 a**/**3 b** and **5** in the presence of DMPO. The formation of DMPO^.^−N_3_ and DMPO^.^−OH radical adducts indicated evolution and trapping of N_3_^.^ and OH^.^ radicals in both cases (Figure 4a, Figure 4c, Figure S33 and Figure S34).

For **3 a**/**3 b**, a steady state total radical adduct concentration was reached after ∼30 min of continuous irradiation and remained almost constant during the period of irradiation (∼3 h) (Figure 4b). For **5**, the total radical adduct concentration peaked after ∼30 min irradiation and then decayed (Figure 4d). Based on our previous work with similar platinum(IV) azido complexes, we attribute the steady radical adduct signal from **3 a**/**3 b** to its high concentration in solution, which saturates the light absorption of the EPR sample at the wavelengths of irradiation.^[8]^

The comparatively low concentration of **5** in the experiment was due to its reduced solubility in H_2_O. DMPO^.^−N_3_: DMPO^.^−OH molar ratios of 90 : 10 and 95 : 5 were determined by spectral simulation for **3 a**/**3 b** and **5**, respectively (Figure 4a and Figure 4c). The DMPO^.^−N_3_ signals quickly disappeared after irradiation ceased, with a lifetime of ∼7 min, demonstrating that radical evolution in aqueous solution can be controlled by blue light irradiation (Figure 4b and Figure S34). The persistance of the DMPO^.^−OH signal is due to the much longer lifetime of that radical adduct in comparison to DMPO^.^−N_3_,^[23]^ rather than due to the evolution of OH^.^ radicals in the dark from **3 a**/**3 b**.

Evolution of OH^.^ radicals from **3 a**/**3 b** in H_2_O is induced by irradiation, evidenced by the initial rise in the DMPO^.^−OH signal (Figure 4b, red trace), while it seems to be light‐independent for 5 ⋅ H_2_O_2_ (Figure 4d, red trace), with the same weak DMPO^.^−OH signal under irradiation as is observed in the dark (Figure S18).

The higher radical molar fraction of DMPO^.^−OH observed for **3 a**/**3 b** could be due to the coexistance of the two forms of the monomeric complex, where **3 a** has two OH ligands per platinum centre that can be potentially released upon irradiation, in comparison to the one OH ligand per platinum in **5** (Scheme 2). Analogous experiments demonstrate that **4**, which is stable in aqueous solution in the absence of light, also evolves predominantly N_3_^.^ radicals under irradiation without undergoing photoreduction, with a smaller fraction of observed DMPO^.^−OH (4 %) than for **3 a**/**3 b** (Figure S36).

ESI‐MS of **4** following 60 min irradiation (452 nmm MeOH) showed [**4**‐N_3_]^+^ at 663.23 *m/z* as the most abundant species, as had been reported previously for **3 a**/**3 b**,^[7]^ with no observable [**4‐**N_3_, OH]^2+^ (anticipated at 324.07 *m/z*) or [**4**‐N_3_, OH]^+^ (anticipated at 646.13 *m/z*) (Figure S37). Since [**4**‐OH]^+^ is already commonly observed as a product of electrospray ionisation both prior and post‐irradiation no conclusions could be drawn as to what proportion of this was produced as a result of OH^.^ ejection or not, but the spectrum was clearly dominated by the [**4**‐N_3_]^+^ species, in agreement with the EPR experiments.

## Conclusions

We have demonstrated that the choice of reaction solvent has a significant effect on the outcome of the cycloaddition reaction between *trans,trans*,*trans*‐[Pt(py)_2_(N_3_)_2_(OH)_2_] (**1**) and 1,4‐diphenyl‐2‐butyne‐1,4‐dione (**2**). Decomposition is observed if the reaction is carried out in MeOH, whereas triazole products **3 a**/**3 b** are formed if the reaction is carried out in MeCN. If residual H_2_O_2_ is present during the cycloaddition reaction, this favours the formation of a peroxide‐bridged intermediate (**5** ⋅ H_2_O_2_) which slowly converts to triazole products **3 a**/**3 b**. If no H_2_O_2_ is present then **3 a**/**3 b** are formed as the major reaction product, with only small amounts of **5** being observed.

The major product **3 a**/**3 b** is stable in MeCN indefinitely, yet suspension in protic solvents causes triazole rearrangement to a new compound **4**, which is then itself stable in solution for at least 12 months. Complex **4** also appears to remain unchanged following re‐isolation and dissolution in MeCN.The rearrangement to **4** can be significantly accelerated (x384) by irradiation with 420 nm light (although slight photodecomposition is also observed), or by modest heating. We are now investigating the effect of irradiation with longer wavelength light on this rearrangement process. Irradiation of complex **4** (440–480 nm) produces predominantly [**4‐**N_3_]^+^ with concommitant azido radical production. Platinum(IV) photoproduct(s) were observed by ESI‐MS and ^195^Pt NMR spectroscopy. EPR experiments confirmed the production of predominantly azido radicals from **3 a**/**3 b**, **4** and **5** ⋅ H_2_O_2_ under blue light (440–480 nm) irradiation in H_2_O, consistent with these findings.

## Experimental Section

**Materials and Methods**. K_2_[PtCl_4_] was purchased from Precious Metals Online. HPLC‐grade solvents and Millipore‐filtered H_2_O were used for the preparation of compounds and purification by HPLC. DMPO (≥98 %) was purchased from Cambridge Bioscience Ltd. All other reagents were purchased from Sigma‐Aldrich or Alfa Aesar and used as received. (IM) indicates use of a nylon syringe filter (pore size 0.2 μM). All manipulations were carried out under reduced lighting and solutions were prepared stored and handled with minimal exposure to light.

**NMR spectroscopy**. Due to the potential photosensitivity of the compounds, amberised NMR spectroscopy tubes (Goss Scientific/Norrell) were used. Spectra were acquired at 298 K unless otherwise stated and processed using Topspin 4.0. All chemical shift (δ) values are given in parts per million and are referenced to residual solvent unless otherwise stated, *J* values are quoted in Hz.^**1**^ **H,** ^**195**^ **Pt and** ^**14**^ **N spectra**: were acquired on a Bruker AVIIIHD 500 MHz (500.13 MHz) equipped with a 5 mm z‐gradient broadband X‐19F/1H BBFO SMART probe, or on a Bruker AVIIIHD 400 nanobay (400.17 MHz). ^**13**^ **C NMR spectra**: were acquired on a Bruker AVII 500 MHz spectrometer equipped with a z‐gradient triple resonance inverse ^1^H/^19^F(^13^C) TXI probe. ^**1**^ **H‐^195^Pt HMBC NMR spectra**: ^195^Pt chemical shifts were externally referenced to K_2_PtCl_6_ in 1.5 mM HCl in D_2_O (δ 0 ppm). ^**1**^ **H NOESY NMR spectra**: run with d8=0.8 (8 scans, 2 h, noesyphsw). ^**14**^ **N NMR spectra**: were acquired with an aring pulse sequence as previously reported.^[19]^ The prescan delay (DE) was 6.5 ms, the 908 pulse length was 17.25 ms and spectra were acquired with a relaxation delay (d1) of 0 s. Spectra were processed to remove the quadrature spike if needed (bc_mod qfil, bcfw 0.02, efp) with an exponential line broadening (lb) of 50. Spectra were externally referenced to a 5 mm tube containing NH_4_Cl (1.5 M) in 1 M HCl, with a D_2_O solution in an internal capillary (δ 0 ppm). **Mass Spectrometry**: low resolution ESI‐MS were obtained with a Waters Micromass LCT Premier XE spectrometer. **HRMS**: obtained with a Thermofisher Exactive Plus with a Waters Acuity UPLC system. **MS/MS experiments**: were performed on an Acuity UPLC in flow injection analysis mode, equipped with a Waters Xevo G25 QTOF. All MS data were processed using MassLynx 4.0. Isotope patterns are predicted using Molecular Weight Calculator http://www.alchemistmatt.com/. **HPLC**: were performed with a Waters Autopurification system, equipped with a Waters X‐Bridge OBD semi‐prep column (5 μm, 19 mm×50 mm), with an injection loop of 1 ml, eluting with H_2_O+0.1 % NH_4_OH (pH 9)/MeCN +0.1 % NH_4_OH. The crude samples (in H_2_O/MeCN) were filtered (nylon, 0.2 μm) and injected in 750 μL aliquots, with mass‐directed purification with an ACQUITY QDa performance mass spectrometer. Analytical HPLC used the same solvents and system, with a Waters X‐Bridge OBD column (5 μm, 4.6 mm×50 mm) and an injection loop of 0.02 ml. Retention times (t_R_) are quoted for the solvent gradient: 0 min (95 % A: 5 % B); 1 min (95 : 5), 7.5 min (5 : 95) on the analytical column. **UV‐visible absorption spectra** were acquired with the Waters HPLC or a UV‐Vis spectrometer (Cary 60 UV‐Vis, Agilent Technologies, Figure S12). **Photochemistry**: samples were irradiated with stirring at a distance of 50 mm from a MiniSun GU10 27 SMD LED bulb with an output centred at 452 nm (see Figure S37) or with a MiniSun UVA bulb. **EPR spectroscopy**: Aqueous samples were prepared as follows. Freshly‐prepared solutions of the complexes, all containing 21 mM DMPO, were loaded into bottom‐sealed quartz capillaries (1.2 mm exterior diameter) using metal needles and plastic syringes. MeCN samples were prepared as follows. DMPO was dissolved in MeCN at a concentration of 21 mM and was degassed in a Schlenk tube by freeze‐pump‐thaw cycles until no further bubbling was observed. The solution was then transferred to a N_2_ glove‐box and used to dissolve complex (previously freeze‐dried) to a Pt concentration of 10.5 mM. This solution was pipetted into a 25 μL glass microcapillary (IntraMark, Blaubrand), and both ends were sealed with *cristaseal* sealing wax (Hawksley). The capillaries were placed in a larger (4 mm external diameter) EPR quartz tubes and inserted into the resonator. Sample volume and position were adjusted so that the length of the sample matched the optical window of the resonator, for an optimal illumination. The EPR experimental setup consisted of a Xe arc lamp (operated at 700 W) coupled to the resonator (Bruker X‐band Super High Sensitivity Probehead) of the EPR spectrometer (X‐band EMXmicro, Brucker) through a liquid waveguide. The light was filtered before entering the waveguide, using a 410 nm long‐pass filter followed by a 440–480 nm band pass filter. Measurements were carried out in continuous‐wave mode, at X‐band frequency (ca. 9.5 GHz) and room temperature, using the following parameters: field sweep of 8 mT, receiver gain of 30 dB, modulation amplitude of 0.1 mT and microwave attenuation of 23.0 dB. Quantification was done using the second integral of the spectra averaged over 100 min of continuous illumination after reaching the steady state, by interpolation of a calibration curve of TEMPO solutions of known concentrations measured in the same conditions as the sample, in the dark. The uncertainty in the radical adduct concentrations was estimated by measuring a TEMPO standard. Simulations were carried out in Matlab®, using a modified EasySpin^[24]^ *garlic* function to allow two species to be fitted simultaneously. For the DMPO^.^−N_3_ adduct, isotropic hyperfine couplings to the nitroxidic nitrogen (a_NO_^N^=1.450 mT), the β‐proton of DMPO (a_β_^H^=1.475 mT) and the α‐nitrogen of the trapped azidyl radical (a_α_^N^=0.314 mT) were considered. For the DMPO^.^−OH adduct, only the first two previous hyperfine couplings were included. An isotropic g‐tensor (g=2.0005) was used in both cases. Radical adduct transients were produced from the spectral peak heights. The DMPO^.^−N_3_ transient was obtained using the two side peaks of each of the four triplets and estimating the height of the central peak as the average of the two side peaks. The DMPO^.^−OH transient was obtained by subtraction of the DMPO^.^−N_3_ transient from the total radical adduct transient.

### Synthetic procedures

#### *Caution*! No problems were encountered during this work, however heavy metal azides are known to be shock sensitive detonators, therefore it is essential that platinum azides compound are handled with care

*Trans, trans, trans*‐[Pt(N_3_)_2_(OH)_2_(py)_2_] (**1**) was synthesised as previously reported. For increased production of **5**, complex **1** was isolated by crystallisation directly from the reaction solution and was not recrystallised before use, retaining residual H_2_O_2_ content.

#### Reaction of 1 with 1,4‐diphenyl‐2‐butyne‐1,4‐dione (2) in MeCN to give complex 5 ⋅ H_2_O_2_ and 3 a/3 b

As previously reported,^[7]^ 1,4‐diphenyl‐2‐butyne‐1,4‐dione (70 mg, 0.299 mmol) and *trans,trans,trans*‐[Pt(N_3_)_2_(OH)_2_(py)_2_] (150 mg, 0.318 mmol) dissolved in MeCN (22 ml) and stirred at 35 °C (48 h) produced complex **3 a**/**3 b** as the major product, and as well as other minor species. These products included dimer **5**, which had not been previously characterised or identified as a dimer, since the mass exceeded the detection limit of the HPLC machine. The solvent volume was reduced to 5 ml *in vacuo*, syringe filtered and purified by HPLC. Complexes **5** ⋅ H_2_O_2_ (t_R_=4.83 min (**31 %**) and **3 a**/**3 b** (t_R_=5.13 min, **13 %**) were isolated and the solvent removed by freeze‐drying to give both as pale yellow solids (see *Figure S2*).

^**1**^ **H NMR (5** ⋅ H_2_O_2_, 500 MHz, *d*_3_‐MeCN) δ: 9.16 (s, br, 1H, H_2_O_2_, OH), 9.14 (dd, ^3^ *J*_HPt_=26.7, ^3^ *J*_HH_=6.7, 4H, H_Pyortho’_), 8.51 (dd, ^3^ *J*_HPt_=26.7, ^3^ *J*_HH_=6.6, 4H, H_Pyortho_), 8.11 (t, ^3^ *J*_HH_=7.6, 2H, H_Pypara’_), 8.06 (d, ^3^ *J*_HH_=8.3, 4H, H_Phortho’_), 7.85 (t, ^3^ *J*_HH_=7.8, 2H, H_Pypara_), 7.68 (t, ^3^ *J*_HH_=7.8, 4H, H_Pymeta’_), 7.59 (t, ^3^ *J*_HH_=7.5, 2H, _Phpara’_), 7.46 (t, ^3^ *J*_HH_=7.8, 4H, _Phmeta’_), 7.22 (t, ^3^ *J*_HH_=6.6, 4H, H_Pymeta_), 7.09 (t, ^3^ *J*_HH_=7.4, 2H, H_Phpara_), 6.95 (t, ^3^ *J*_HH_=7.8, 4H, H_Phmeta_), 6.75 (d,^3^ *J*_HH_=8.3, 4H, H_Phortho_). ^**13**^ **C NMR** (**5** ⋅ H_2_O_2,_ 151 MHz, *d*_3‐_MeCN) δ: 187.9 (q, CO), 153.3 (q, triazole), 151.6 (q, triazole, ^2^ *J*_CPt_ 43.8 Hz), 150.42 (Py_ortho_), 150.37 (Py_ortho’_), 143.1 (q, Ph_ipso_), 143.0 (Py_para’_), 142.8 (Py_para_), 138.6 (q, Ph’_ipso_), 133.8 (Ph_para’_), 131.3 (Ph_ortho’_), 129.1 (Ph_meta’_), 127.2 (t, ^3^ *J*_CPt_= 27.2, Py_meta’_), 127.0 (t, ^3^ *J*_CPt_= 26.2, Py_meta_), 128.8 (Ph_para_), 128.6 (Ph_meta_), 126.4 (Ph_ortho_), 112.1 (q, C−OH).

#### Characterisation of 3 a/3 b in *d*_4_‐MeOH (freshly prepared) before rearrangement

^**195**^ **Pt NMR** (107 MHz, *d*_4_‐MeOH) δ: 832 and 784.

**IR** ν cm^−1^ (*d*_4_‐MeOH): 3418.25(br), 2505.31(br), 2360.65, 2342.09, 2047.76 (sharp, ν_asym_N_3_), 1652.13, 1613.86, 1596.93, 1576.75, 1458.93, 1400.85, 1258.62, 1213.35, 1077.10, 1022.18, 946.22, 898.43, 768.63, 690.11. Additional characterisation of **3 a**/**3 b** is as previously described.^[7]^

#### Characterisation of complex 4 following rearrangement in *d*_4_‐MeOH

^**1**^ **H NMR** (**4**, 400 MHz, *d*_4_‐MeOH) δ: 9.05 (d, ^3^ *J*_HPt_=25, ^3^ *J*_HH_=6, 2H, H_Pyortho’_), 8.54 (d, ^3^ *J*_HPt_=25, ^3^ *J*_HH_=6, 2H, H_Pyortho_), 8.26 (dd, ^3^ *J*_HH_=6, 1H, H_Pypara’_), 7.97 (dd,^3^ *J*_HH_=6, 1H, H_Pypara_), 7.93 (d, ^3^ *J*_HH_=6, 2H, H_Phortho’_), 7.81 (dd, ^3^ *J*_HH_=7, 2H, H_Pymeta’_), 7.57 (t, ^3^ *J*_HH_=7, 1H, H_Phpara’_), 7.43 (dd, 2H, ^3^ *J*_HH_=7, H_Phmeta’_), 7.33 (dd, 2H, H_Pymeta_), 7.04 (dd, 1H, ^3^ *J*_HH_=7, H_Phpara_), 6.91 (t, 2H, ^3^ *J*_HH_=7, H_Phmeta_), 6.71 (d, 2H, ^3^ *J*_HH_=6, H_Phortho_).

^**13**^ **C NMR** (**4**, 126 MHz, *d*_4_‐MeOH) δ: 188.0 (q, C_PhCO_), 152.0 (q, C_triazole_), 151.3 (C_Pyortho’_), 150.7 (C _Pyortho_), 145.3 (q, C_ipsoPh_), 143.8 (C_Pypara’_), 143.6 (q, C_triazole_), 143.4 (C_Pypara_), 138.8 (q, C_ipsoPh’_), 134.1 (C_Phpara’_), 131.2 (C_Phortho’_), 129.9 (*_impurity_), 129.2 (C_Phmeta’_), 128.8 (C_Phmeta_), 128.6 (C_Phpara_), 128.1 (^3^ *J*_13C195Pt_= 26, C_Pymeta’_), 127.6 (^3^ *J*_13C195Pt_= 26, C_Pymeta’_), 126.3 (C_Phortho_), 108.4 (q, C_PhC‐OX_).

^**195**^ **Pt NMR** (**4**, 107 MHz, *d*_4_‐MeOH) δ: 824.

**IR** v cm^−1^ (**4**, *d*_4_‐MeOH): 3391.11 (br), 2508.44 (br), 2048.63 (sharp, ν_asym_N_3_), 1654.70, 1613.38, 1597.02, 1460.08, 1405.16, 1254.93, 1213.32, 1106.53, 1013.34, 934.54, 894.13, 768.44, 691.38.

**ESI‐MS** (**4**, MeOH) *m/z*: (M=*trans,trans,trans*‐[Pt(N_3_)(C_16_H_10_N_3_O_2_)(OH)_2_(py)_2_]): 705.71 ([M+H]^+^ C_26_H_23_N_8_O_4_Pt, calcd 706.15), 687.83 ([M−OH]^+^ C_26_H_21_N_8_O_3_Pt, calcd 688.13).

Deposition Number 2008628 (for **5**) contains the supplementary crystallographic data for this paper. These data are provided free of charge by the joint Cambridge Crystallographic Data Centre and Fachinformationszentrum Karlsruhe Access Structures service www.ccdc.cam.ac.uk/structures.

## Conflict of interest

The authors declare no conflict of interest.

## Supporting information

As a service to our authors and readers, this journal provides supporting information supplied by the authors. Such materials are peer reviewed and may be re‐organized for online delivery, but are not copy‐edited or typeset. Technical support issues arising from supporting information (other than missing files) should be addressed to the authors.

Supplementary
