# Supplementary material for: Solvent‐Dependent Reactivity and Photochemistry of Dinuclear and Mononuclear Platinum(IV) Azido Triazaolato Complexes
Source: Eur J Inorg Chem. 2021 Mar 16;2021(14):1397–404. doi: 10.1002/ejic.202100041 (PMC8251955; doi:10.1002/ejic.202100041)
Supplement: Supplementary file 1 — Supplementary [file EJIC-2021-1397-s001.pdf]

European Journal of Inorganic Chemistry

Supporting Information

Solvent-Dependent Reactivity and Photochemistry of Dinuclear and Mononuclear Platinum(IV) Azido Triazaolato Complexes

Kezi Yao, Arnau Bertran, Jacques Morgan, Charlotte Greenhalgh, Katharina Edkins, Alice M. Bowen, and Nicola J. Farrer*

Author Contributions

K.Y. Conceptualization:Supporting; Data curation:Supporting; Formal analysis:Supporting; Writing – review & editing:Supporting
A.B. Data curation:Supporting; Formal analysis:Supporting; Methodology:Supporting; Writing – review & editing:-Supporting
C.G. Data curation:Supporting
K.E. Conceptualization:Supporting; Data curation:Supporting; Writing – review & editing:Supporting

Contents

HPLC traces of products 2
Characterisation of complex **5** 4
IR spectroscopy of **5**.H_2O_2 8
ESI-MS of **5**.H_2O_2 9
ESI-MS/MS of **5**.H_2O_2 10
NMR Spectroscopy of **5**.H_2O_2 11
EPR spectroscopy of 5.H_2O_2 20
Conversion of **3a/3b** to **4** in *d*$_4$-MeOH 23
Conversion of **3a/3b** to **4** monitored by ^{1}H NMR spectroscopy 24
ESI-MS of complex 4 25
^{1}H NMR spectroscopy of complex 4 26

HPLC traces of products

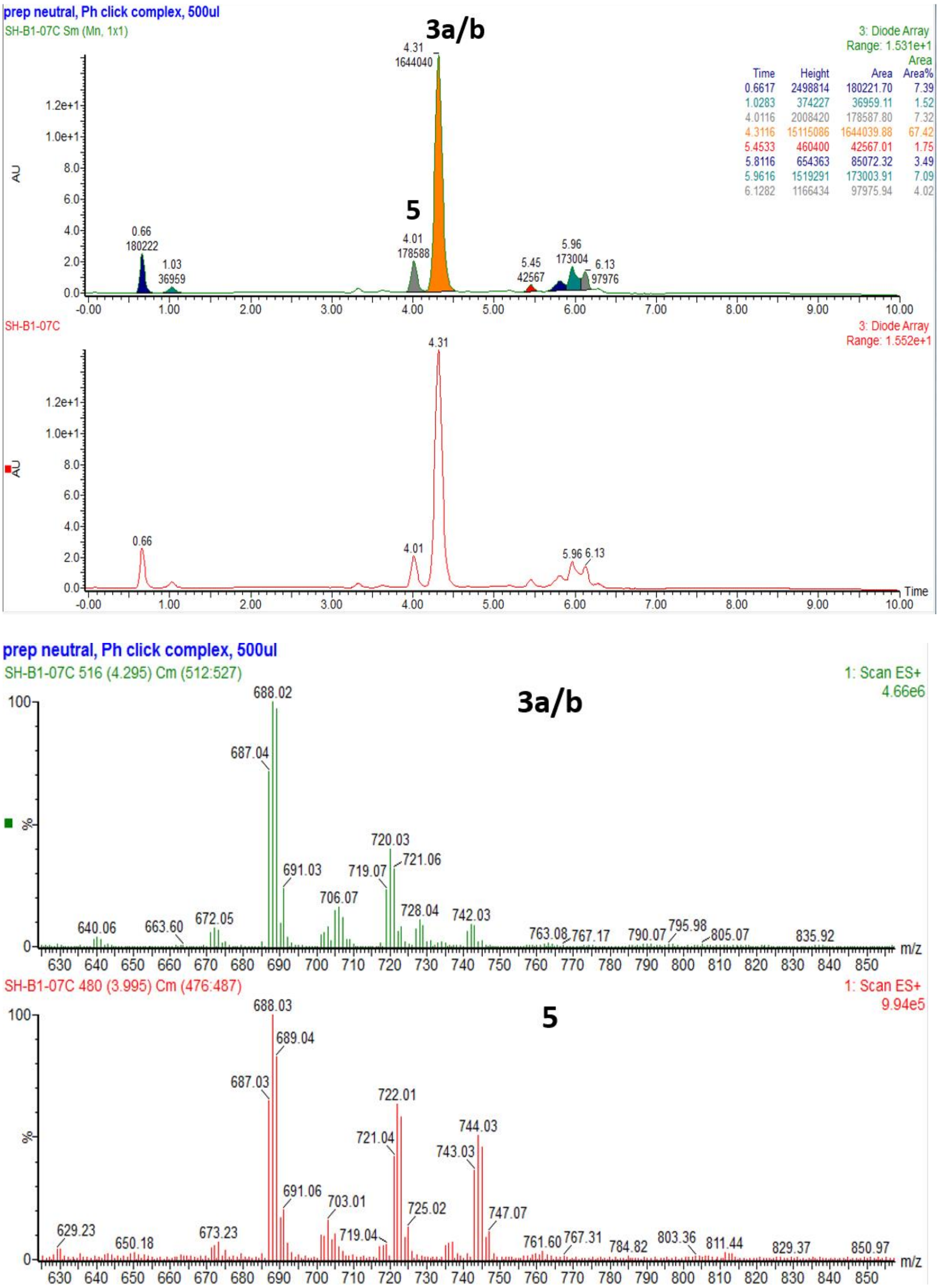

Figure S1. *Top:* Comparative HPLC trace of crude mixture from reaction between complex **1** and **2** showing product distribution if H_2O_2 <u>is not</u> present in the starting material. *Bottom:* extracted ESI-MS spectra of **5** and **3a/b** from the HPLC trace. (*Trace reproduced here from ref.* [1] *for comparison*).

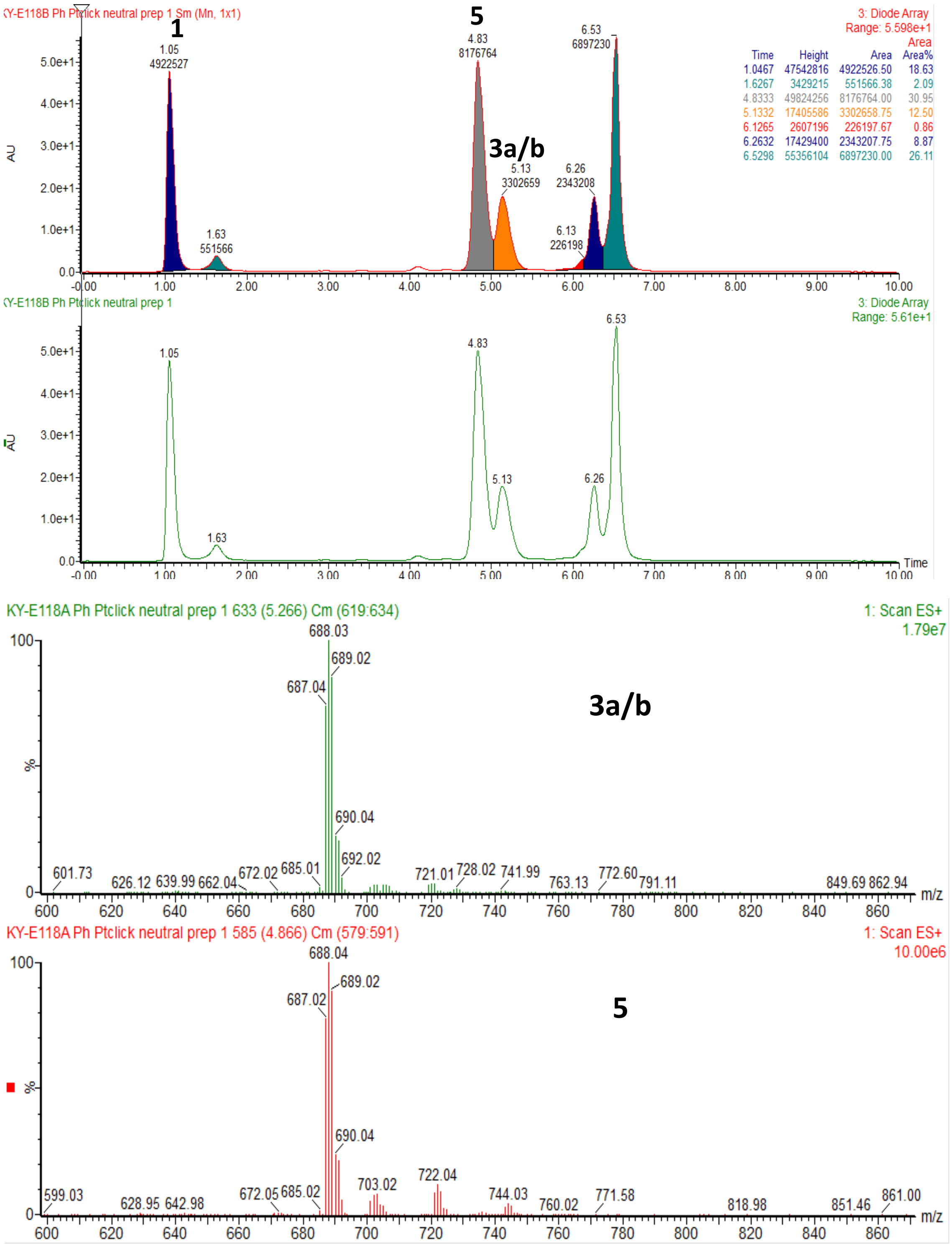

Figure S2. *Top:* HPLC trace of crude mixture from reaction reported here between complex **1** and **2** showing product distribution if H_2O_2 is present in the starting material. Key compounds: t_R = 4.83 min (**5, 31%**), 5.13 min (complex **3a/3b, 13%**). Unreacted starting material (**1**) is detected at 1.05 min. Relative product distributions are calculated by relative UV-Vis spectral absorbance integrated over the range 210 nm – 400 nm. *Bottom:* extracted ESI-MS spectra of **5** and **3a/b** from the HPLC trace.

Characterisation of complex **5**

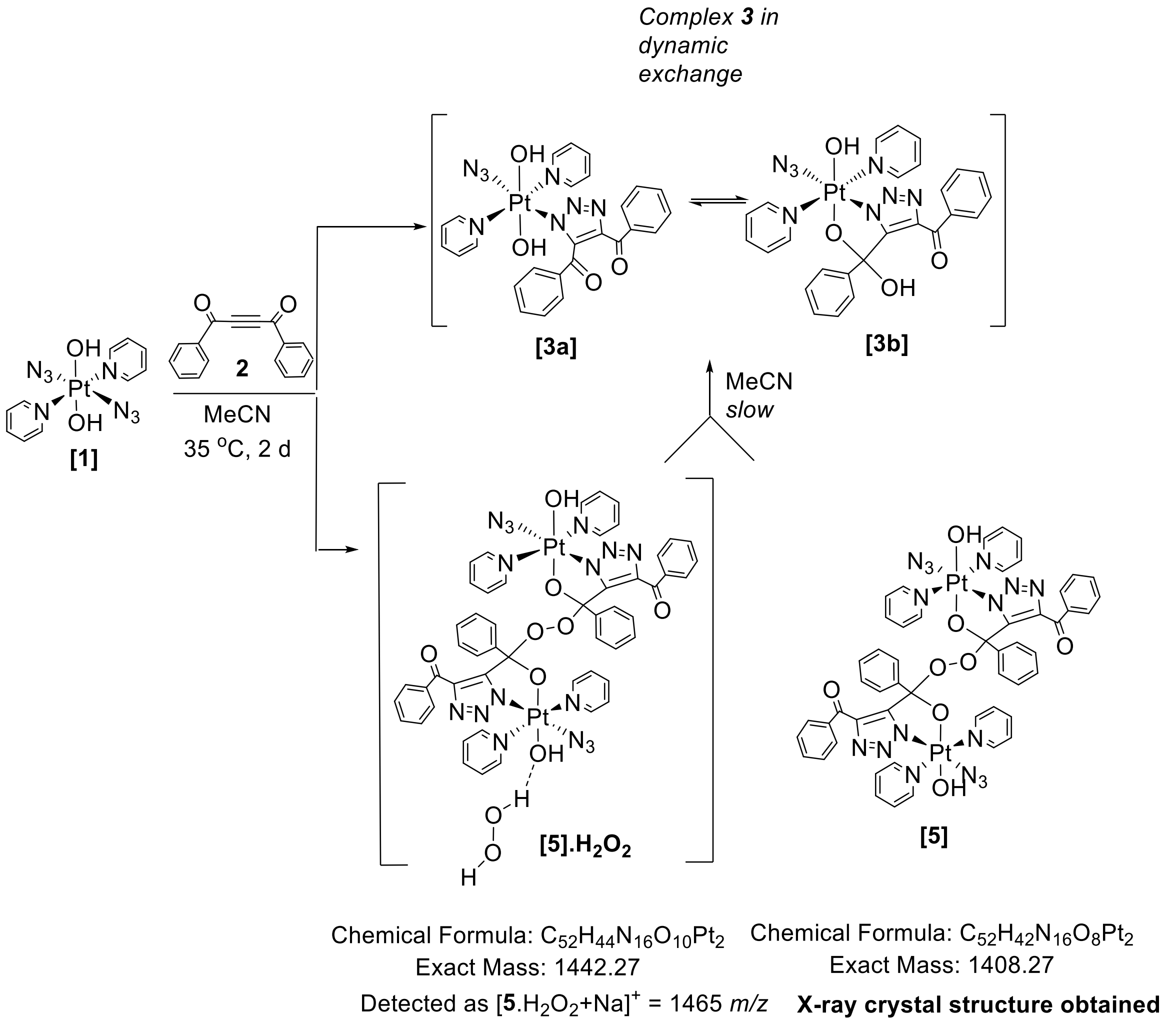

Figure S3. Synthesis of complex **5** from reaction between **1** and **2** in MeCN and putative structure of **5**.H_2O_2, which is consistent with the analytical data.

X-ray crystallography of 5, co-crystallised with MeCN.

Single crystal suitable for diffraction measurement were picked and dropped directly into perfluoro polyether oil. The chosen crystals were mounted on MiTeGen sample loops and placed directly onto the diffractometer. Date were collected on an Agilent Supernova diffractometer using Mo Kα radiation (0.71073 Å). The crystals were kept at room temperature. Data reduction was performed with the Crysalis programme, structure solution using olex2.solve and refinement with olex2.refine.[2] All non-hydrogen atoms were picked from the electron density maps and refined anisotropically. Hydrogen atoms were placed in geometrically optimised positions and refined as riding models.

Crystal Data for $C_{54.5}H_{46.5}N_{17.5}O_8Pt_2$ [Complex **5**.MeCN) (*M* =1464.77 g/mol): triclinic, space group P-1 (no. 2), *a* = 9.8284(3) Å, *b* = 14.7485(8) Å, *c* = 20.9424(8) Å, *α* = 80.340(4)°, *β* = 83.445(3)°, *γ* = 84.553(4)°, *V* = 2964.2(2) $Å^3$, *Z* = 2, *T* = 298 K, μ(MoKα) = 4.781 mm^{-1}, *Dcalc* = 1.641 g/cm^3, 19786 reflections measured (6.744° ≤ 2Θ ≤ 52°), 11536 unique (R_{int} = 0.0427, R_{sigma} = 0.0866) which were used in all calculations. The final R_1 was 0.0462 (I > 2σ(I)) and wR_2 was 0.1098 (all data).

**Table S4**. Crystal data and structure refinement for **18KE09_2**.	
Identification code	18KE09_2
Empirical formula	$C_{54.5}H_{46.5}N_{17.5}O_8Pt_2$
Formula weight	1464.77
Temperature/K	298
Crystal system	triclinic
Space group	P-1
a/Å	9.8284(3)
b/Å	14.7485(8)
c/Å	20.9424(8)
α/°	80.340(4)
β/°	83.445(3)
γ/°	84.553(4)
Volume/$Å^3$	2964.2(2)
Z	2
$\rho_{calc}g/cm^3$	1.641

μ/mm^{-1}	4.781
F(000)	1432.0
Crystal size/mm^3	0.808 × 0.1 × 0.05
Radiation	MoKα (λ = 0.71073)
2Θ range for data collection/°	6.744 to 52
Index ranges	-12 ≤ h ≤ 10, -18 ≤ k ≤ 17, -21 ≤ l ≤ 25
Reflections collected	19786
Independent reflections	11536 [R_{int} = 0.0427, R_{sigma} = 0.0866]
Data/restraints/parameters	11536/24/775
Goodness-of-fit on F^2	1.049
Final R indexes [I>=2σ (I)]	R_1 = 0.0462, wR_2 = 0.0949
Final R indexes [all data]	R_1 = 0.0749, wR_2 = 0.1098
Largest diff. peak/hole / e $Å^{-3}$	1.86/-1.61

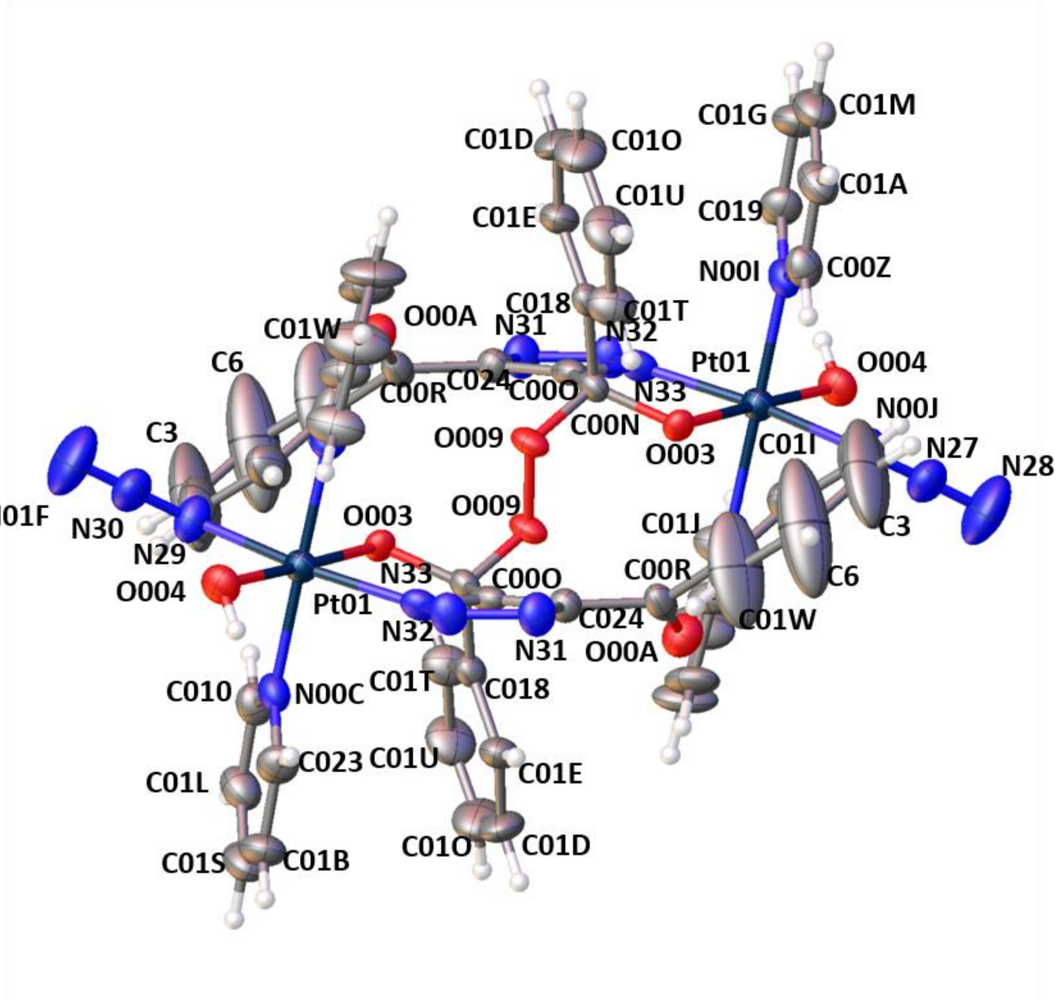

Figure S4. Fully labeled X-ray crystallographic OLEX2 structure of the peroxide dimer **5** with thermal ellipsoids displayed at 50%.

IR spectroscopy of **5**.H_2O_2

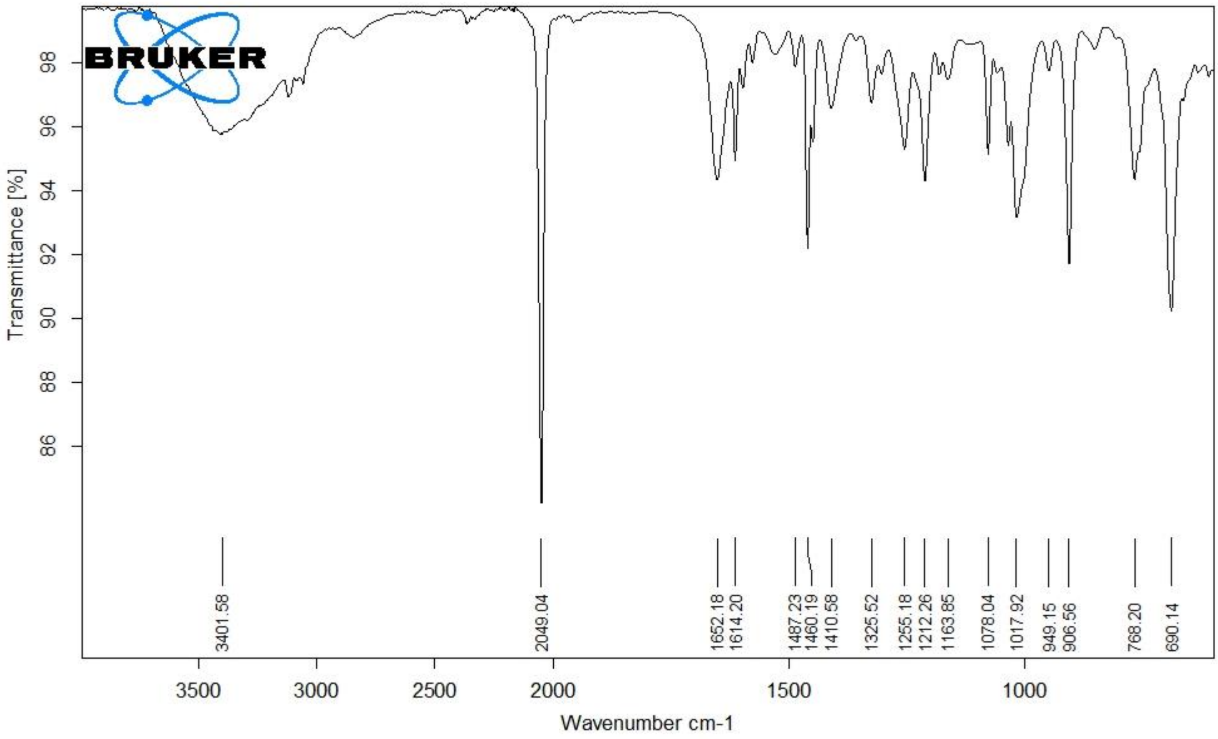

Figure S5. IR spectrum of complex **5**.H_2O_2 (MeOH).

ESI-MS of **5**.H_2O_2

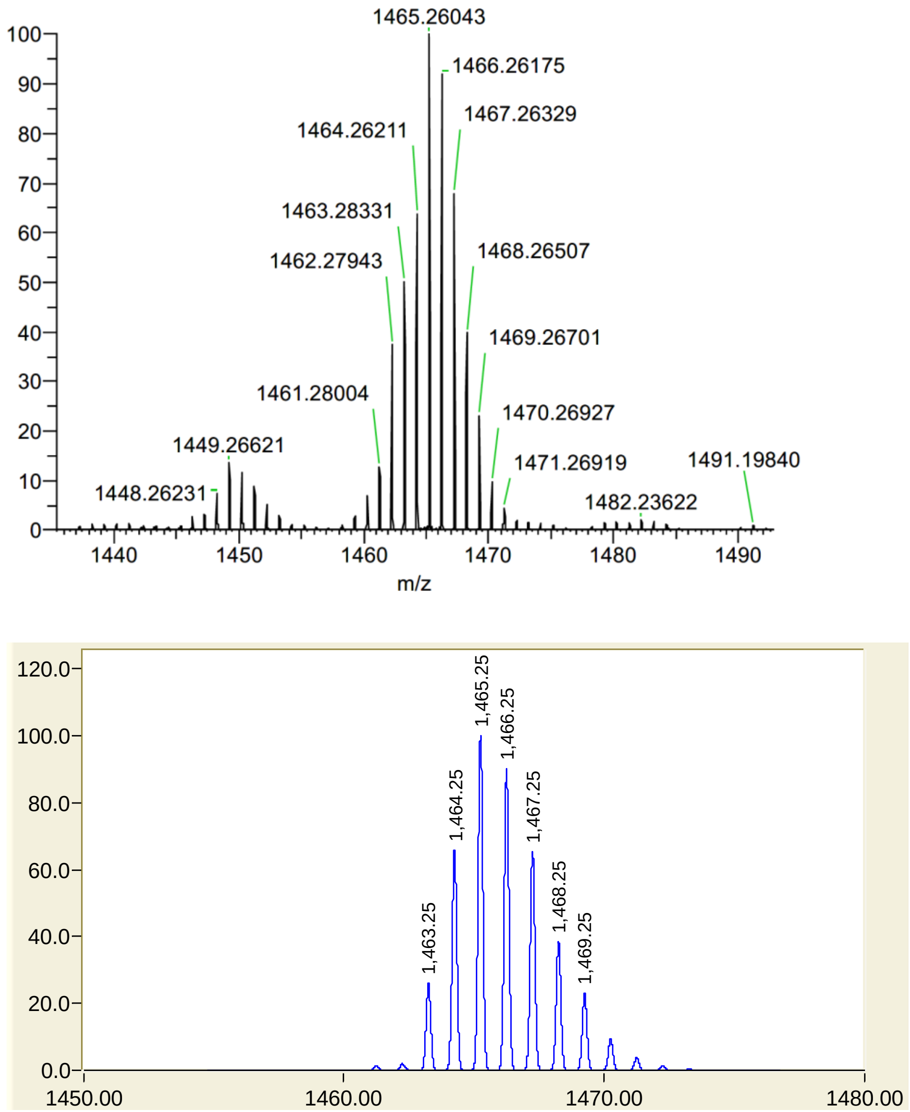

Figure S6. HRMS of complex [**5**.H_2O_2 +Na]$^+$ ($C_{52}H_{44}N_{16}NaO_{10}Pt_2$); *top*: 1465.2604 *m/z* found; *bottom*: 1465.2522 *m/z* calculated (5.6 ppm error).

ESI-MS/MS of **5**.H_2O_2

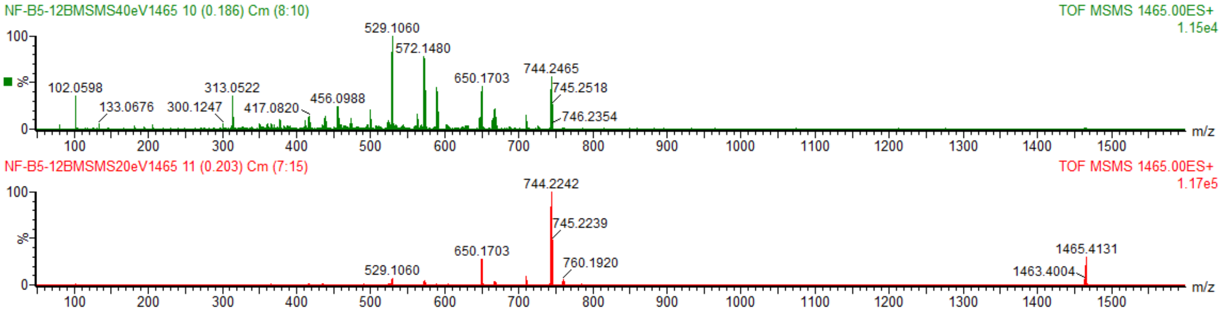

Figure S7. MS/MS fragmentation of complex [**5a**.H_2O_2+Na]$^+$ at 1465 *m/z* in MeCN. *Top*: 40 eV; *Bottom*: 20 eV: main fragment [Pt(OH)$_2$(N$_3$)(triazole)(py)$_2$O+Na]$^+$ (calcd. $C_{26}H_{22}N_8O_5PtNa$) at 744.22 *m/z*.

NMR Spectroscopy of **5**.H_2O_2

^{195}Pt NMR spectroscopy

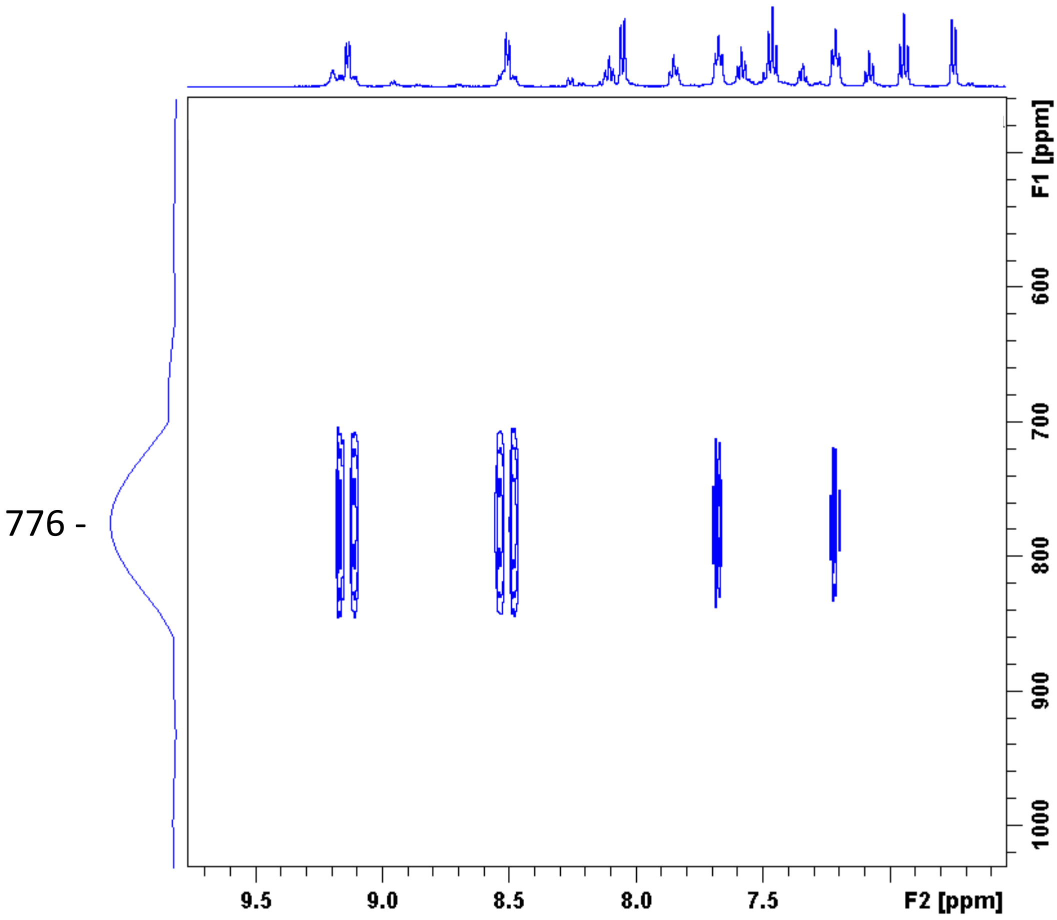

Figure S8. ^{195}Pt NMR spectrum (107 MHz, d_3-MeCN) δ: 776.

^{1}H NMR spectroscopy

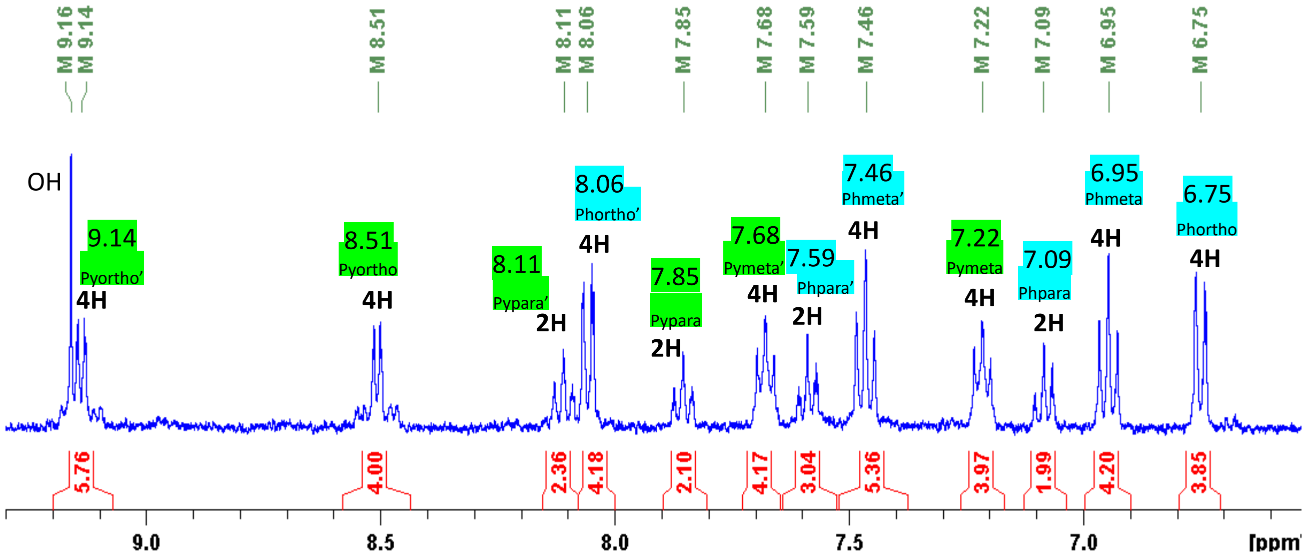

Figure S9. ^{1}H NMR spectrum (500 MHz, d_3-MeCN) of **5**.H_2O_2.

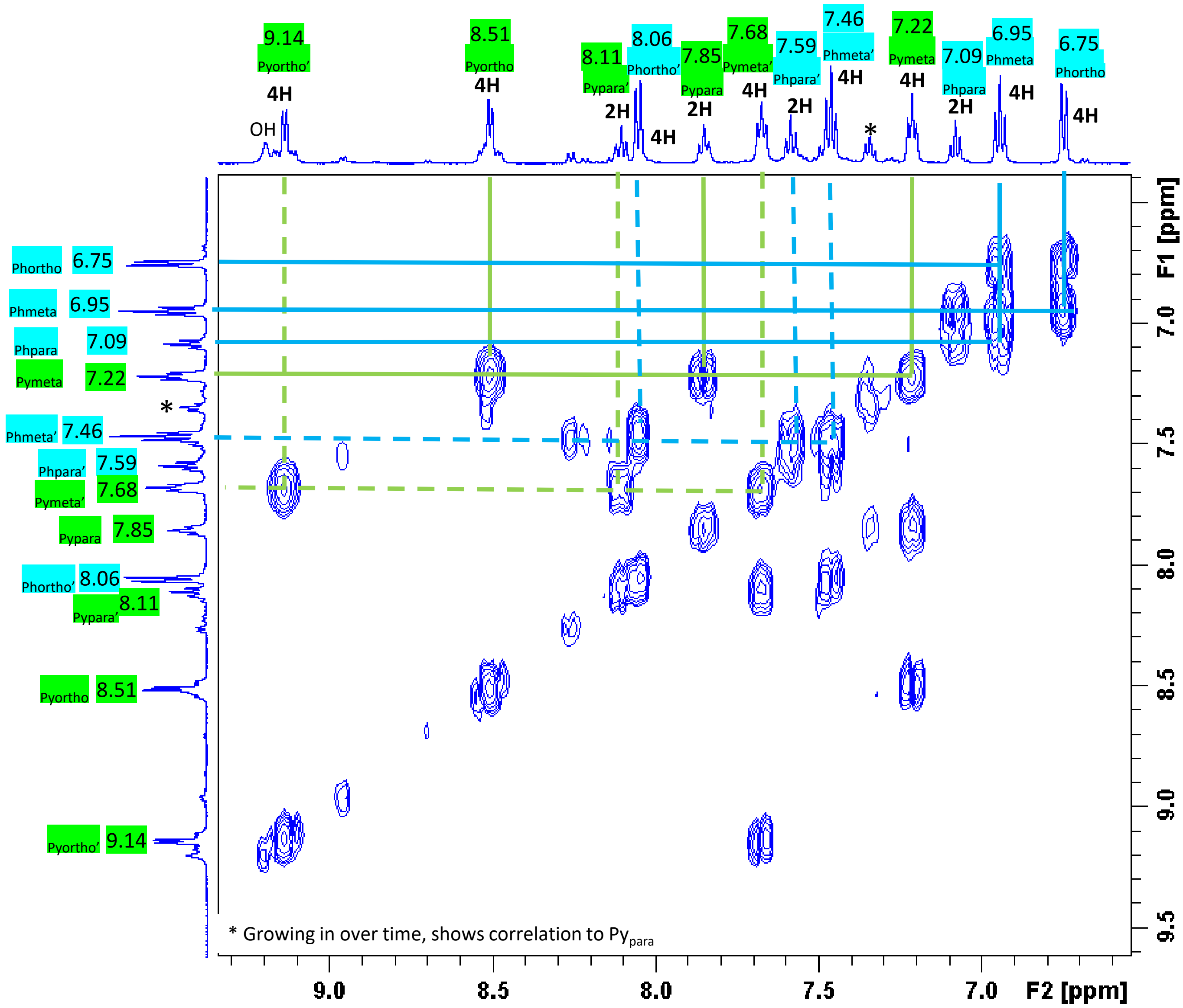

Figure S10. ^{1}H NMR COSY spectrum (d_3-MeCN) of **5**.H_2O_2.

^{1}H NMR assignment:

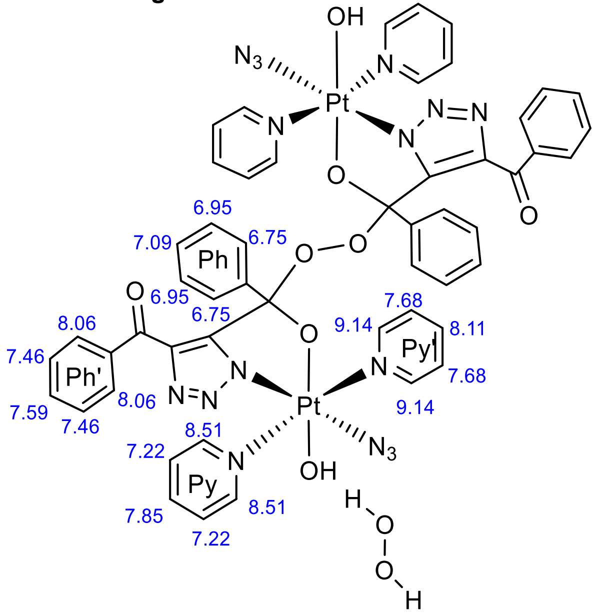

[5].H_2O_2

^{13}C NMR assignment:

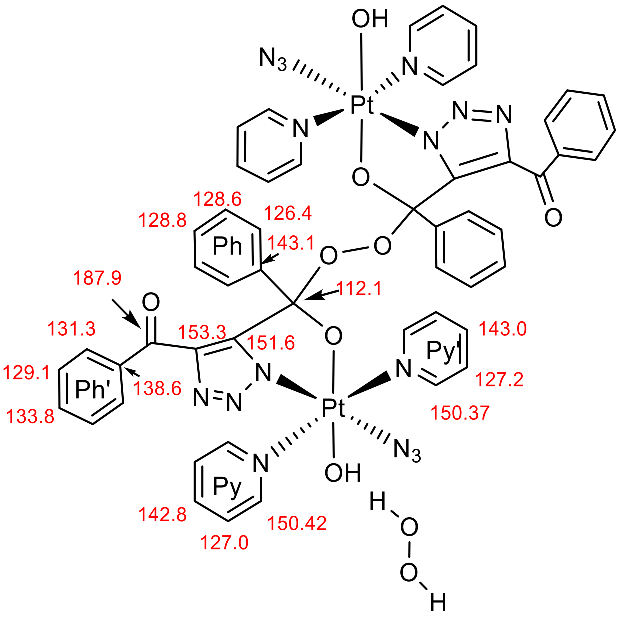

[5].H_2O_2

Figure S11. Labelled structure of **5**.H_2O_2 with ^{1}H and ^{13}C NMR spectral assignments.

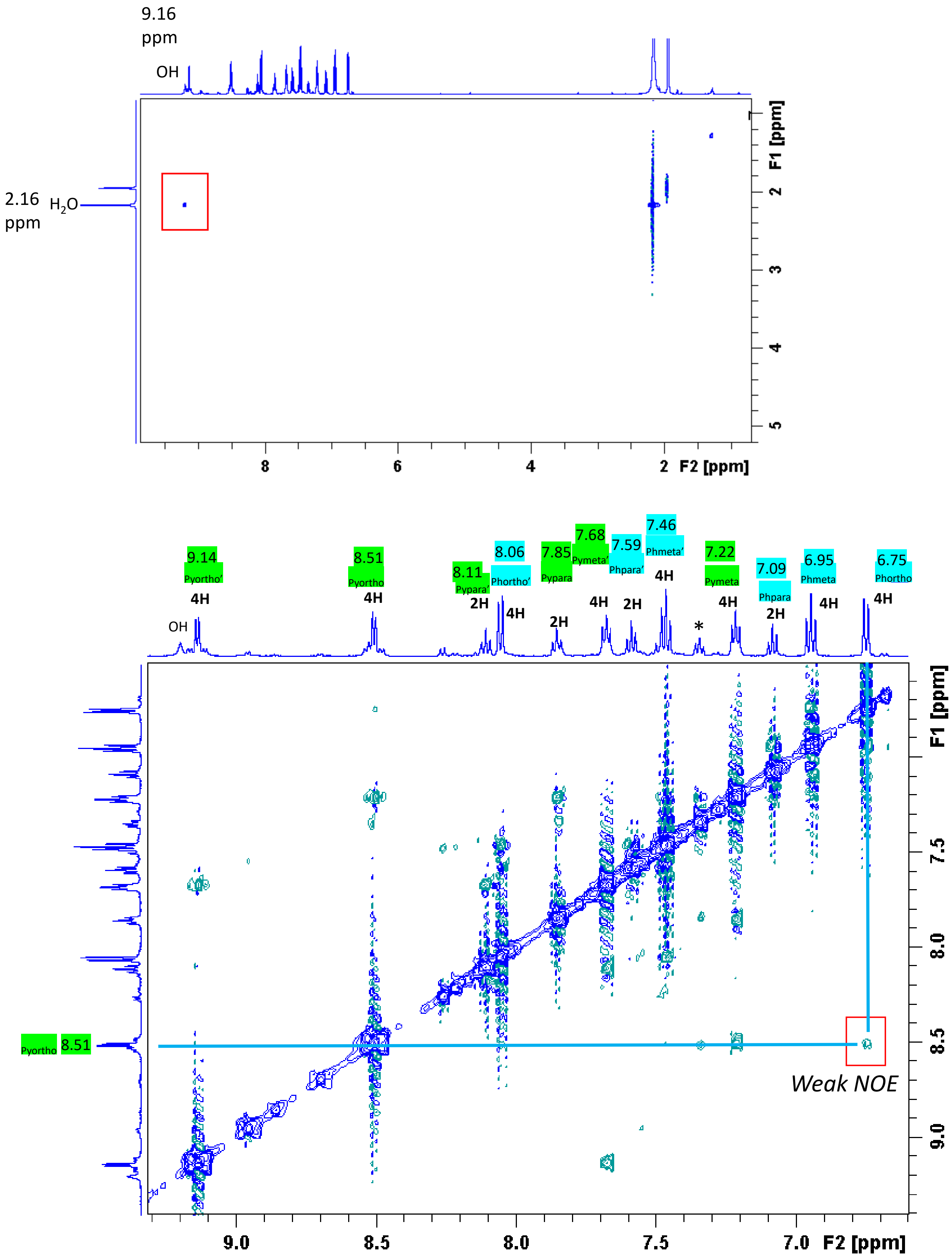

Figure S12. ^1H NOESY NMR spectra (d_3-MeCN) of **5**.H_2O_2.

^{13}C NMR spectroscopy

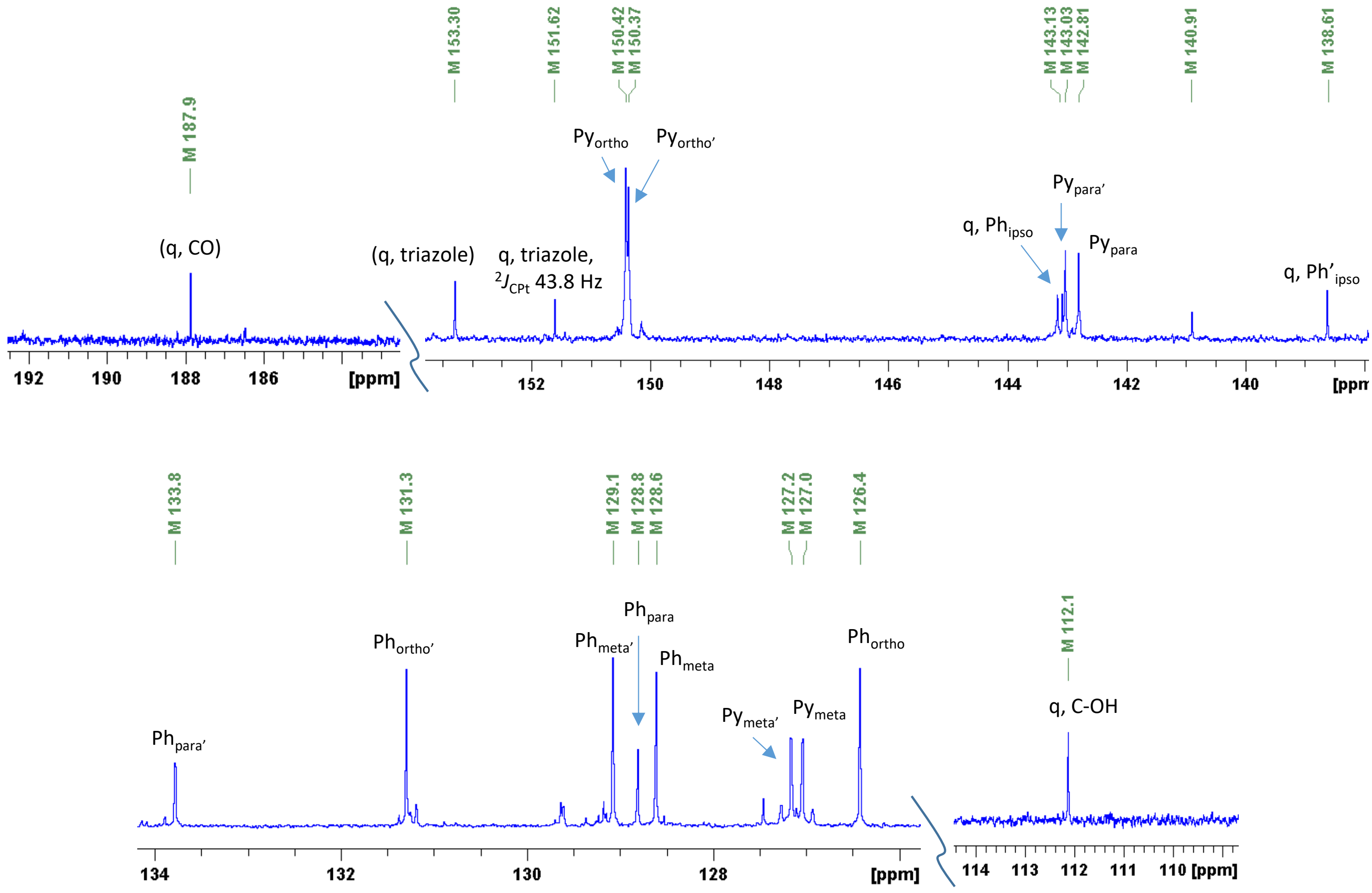

Figure S13. ^{13}C NMR spectrum (d_3-MeCN) of **5**.H_2O_2

^{1}H – ^{13}C HSQC spectra

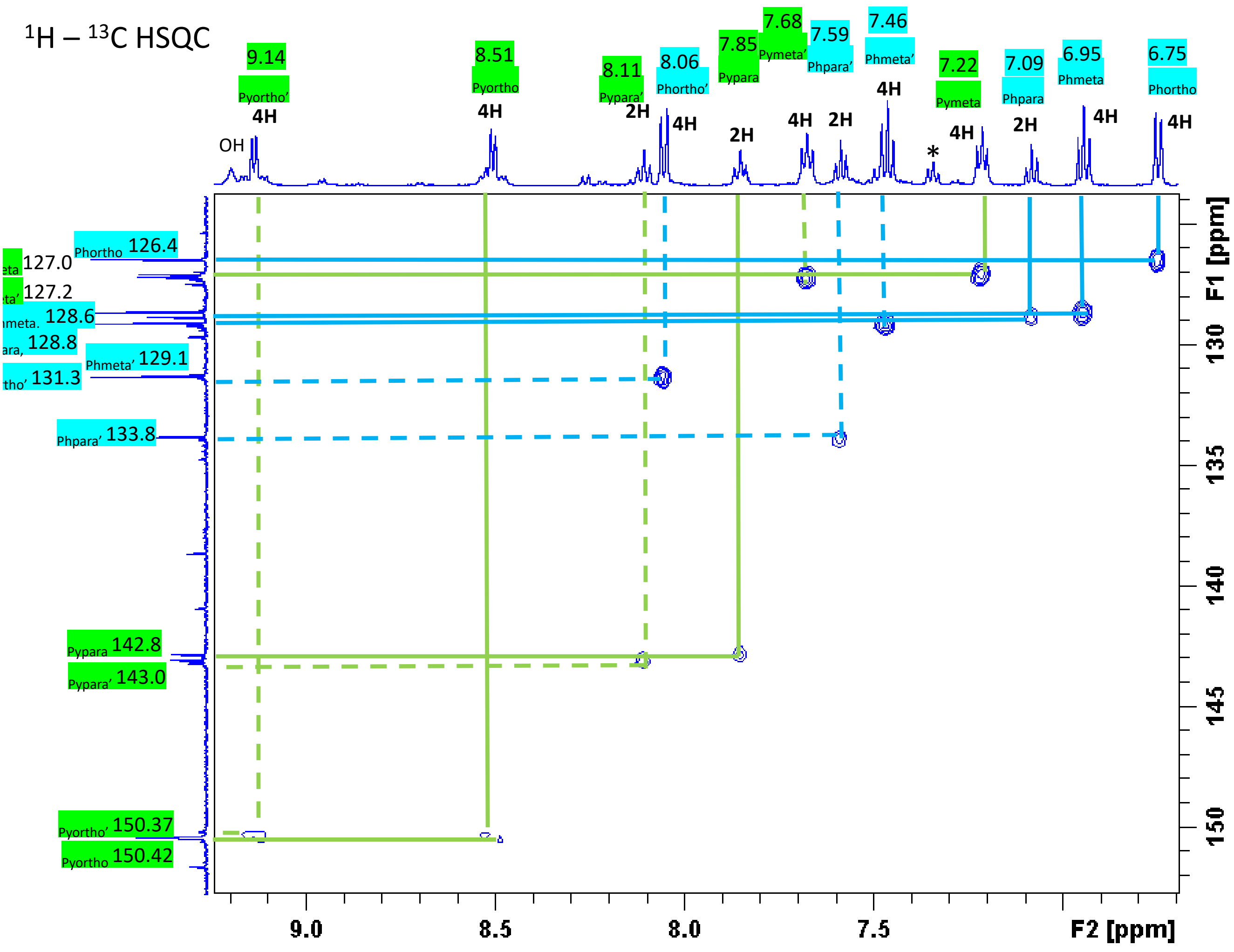

Figure S14. ^{1}H - ^{13}C HSQC NMR spectra (d_3-MeCN) of **5**.H_2O_2.

^1H – ^{13}C HMBC spectra

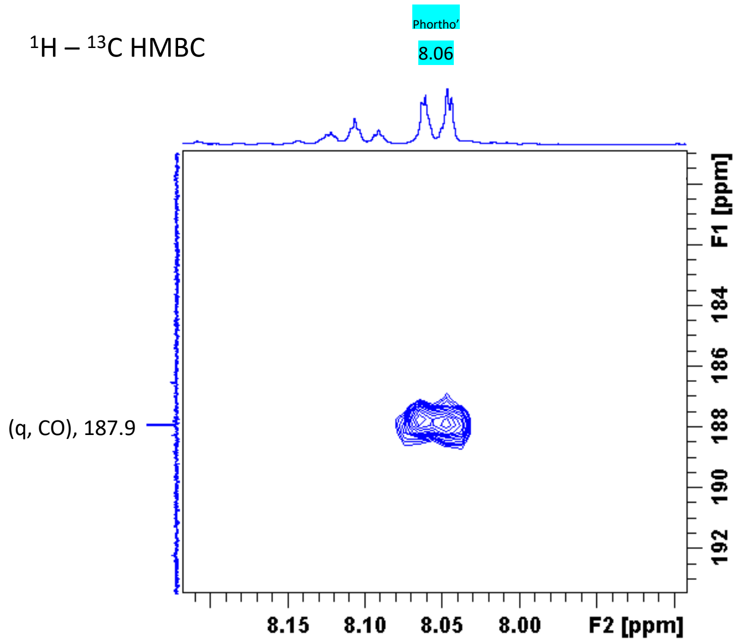

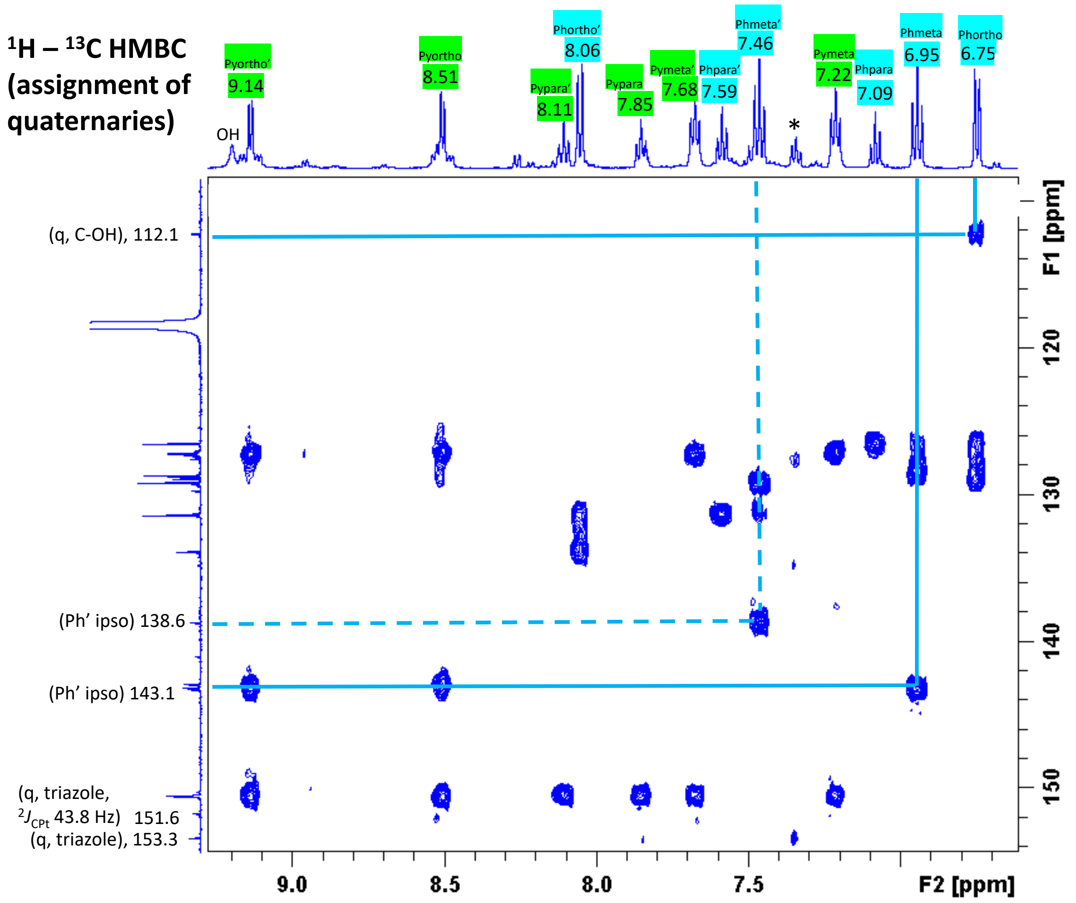

Figure S15. ^1H - ^{13}C HMBC NMR spectra (d_3-MeCN) of **5**.H_2O_2

UV-vis spectra of 5.H_2O_2

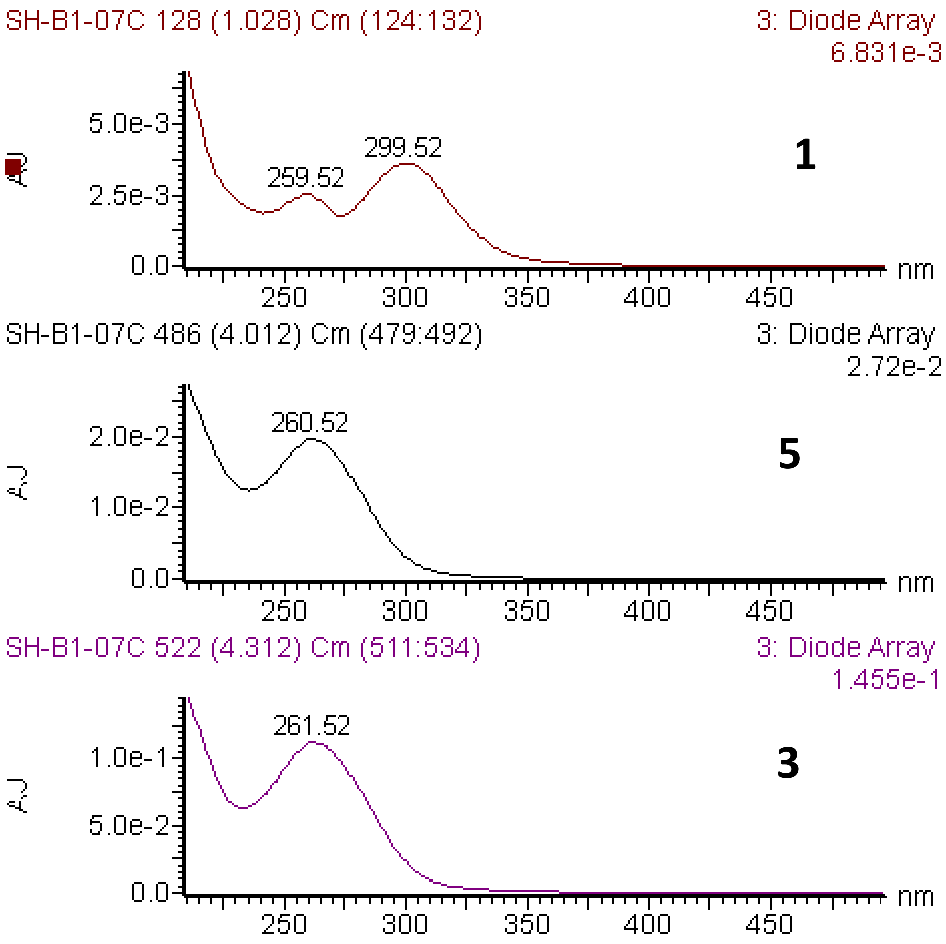

Figure S16. UV-Vis (MeCN/H_2O) of **5**.H_2O_2 in comparison to **3a/3b** and **1**.

EPR spectroscopy of 5.H_2O_2

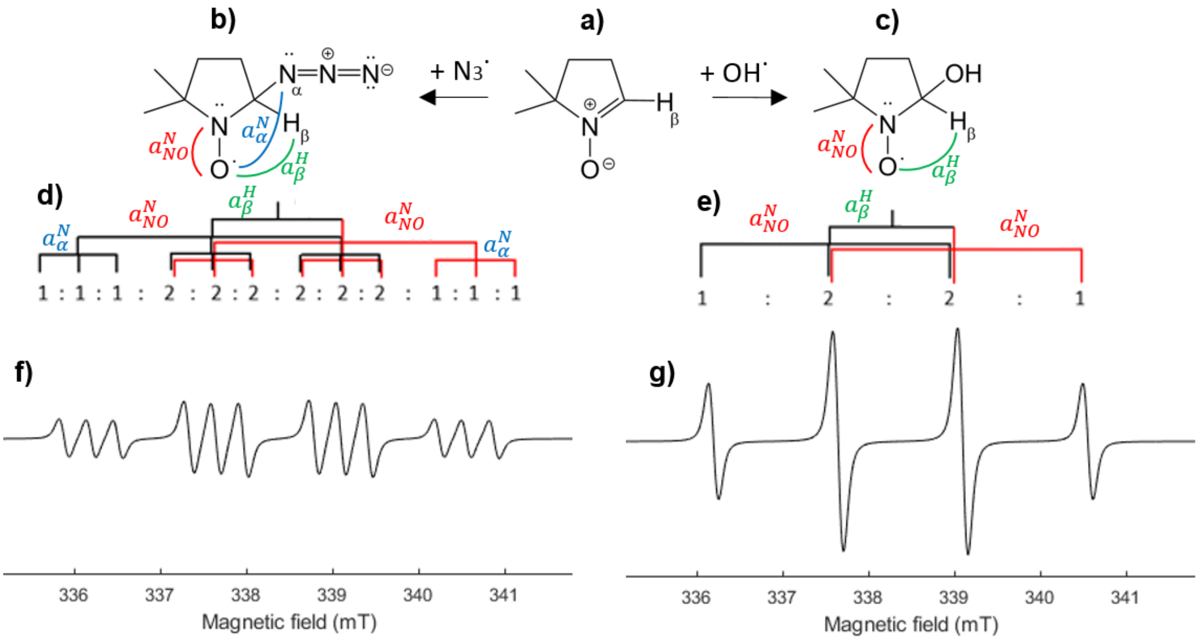

Figure S17. Spin trapping of $N_3^{\cdot}$ and $OH^{\cdot}$ radicals with 5,5-Dimethyl-1-pyrroline N-oxide (DMPO). Chemical structures of (a) DMPO, (b) DMPO$^{\cdot}$-N_3 radical adduct and (c) DMPO$^{\cdot}$-OH radical adduct, where the hyperfine coupling constants of the unpaired electron to the adjacent nuclei have been indicated as a_H (green), a_{NO}^N (red) and a_N (blue). (d,e) Hyperfine splitting diagrams indicating the relative peak intensity. (f,g) Corresponding room temperature X-band cw-ESR spectra simulated using Matlab *EasySpin*.[2] Note that the apparent multiplicity of the spectra is reduced due to the fact that $a_H \approx a_{NO}^N$.

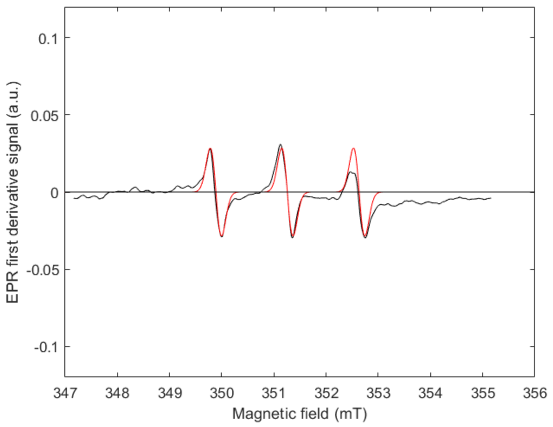

Figure S18. Cw-EPR spectrum of a solution of **5**.H_2O_2 (10.5 mM Pt) and DMPO (21 mM) in degassed MeCN, which had been kept in the dark for 19 d (black). The spectrum was averaged for 75 min in the dark. The simulation (red) was done considering only the hyperfine coupling to one nitrogen (a^N = 1.37 mT).

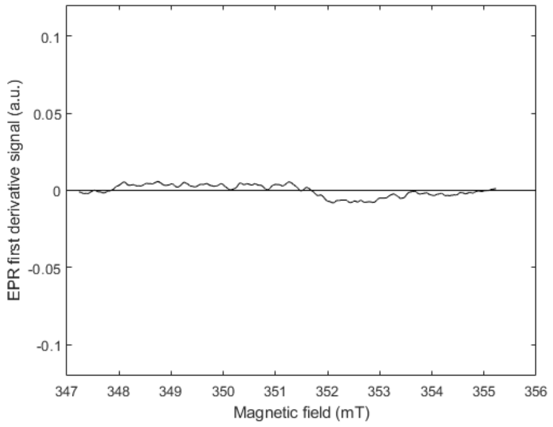

Figure S19. Cw-EPR spectrum of a solution of **3a/3b** (10.5 mM Pt) and DMPO (21 mM) in degassed MeCN, which had been kept in the dark for 9 d. The spectrum was averaged for 75 min in the dark.

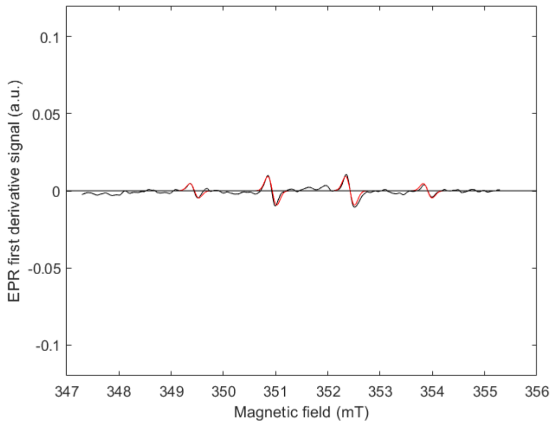

Figure S20. Cw-EPR spectrum of a solution of **5**.H_2O_2 (< 1 mM Pt) and DMPO (21 mM) in water, which had been kept in the dark for 5 d (black). The spectrum was averaged for 75 min in the dark. The simulation (red) was done considering the hyperfine couplings expected for the DMPO•-OH radical adduct ($a_{NO}^{N} = a_{\beta}^{H} = 1.49$ mT).

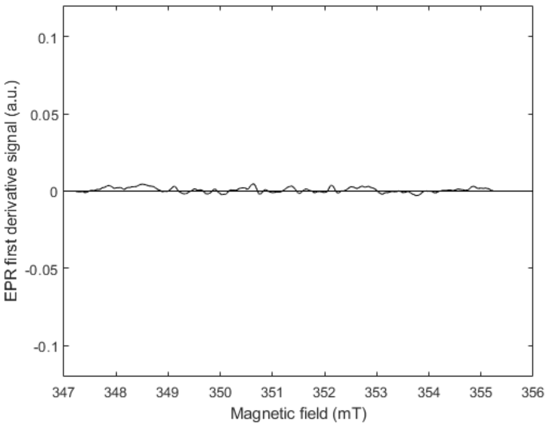

Figure S21. Cw-EPR spectrum of a solution of **3a/3b** (10.5 mM Pt) and DMPO (21 mM) in water, which had been kept in the dark for 7 days. The spectrum was averaged for 75 min in the dark.

Conversion of **3a/3b** to **4** in d_4-MeOH

^{1}H NMR assignment:

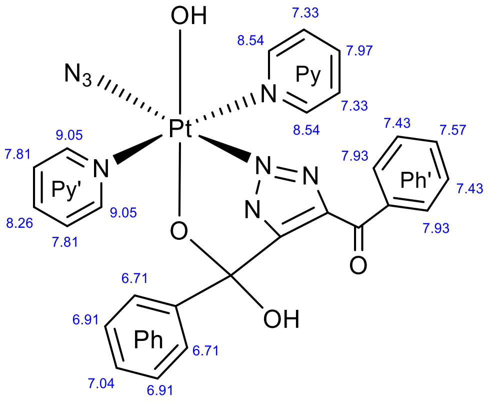

[4]

^{13}C NMR assignment:

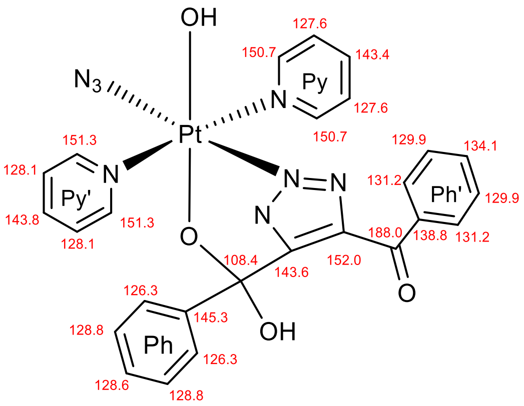

[4]

Conversion of **3a/3b** to **4** monitored by ^{1}H NMR spectroscopy

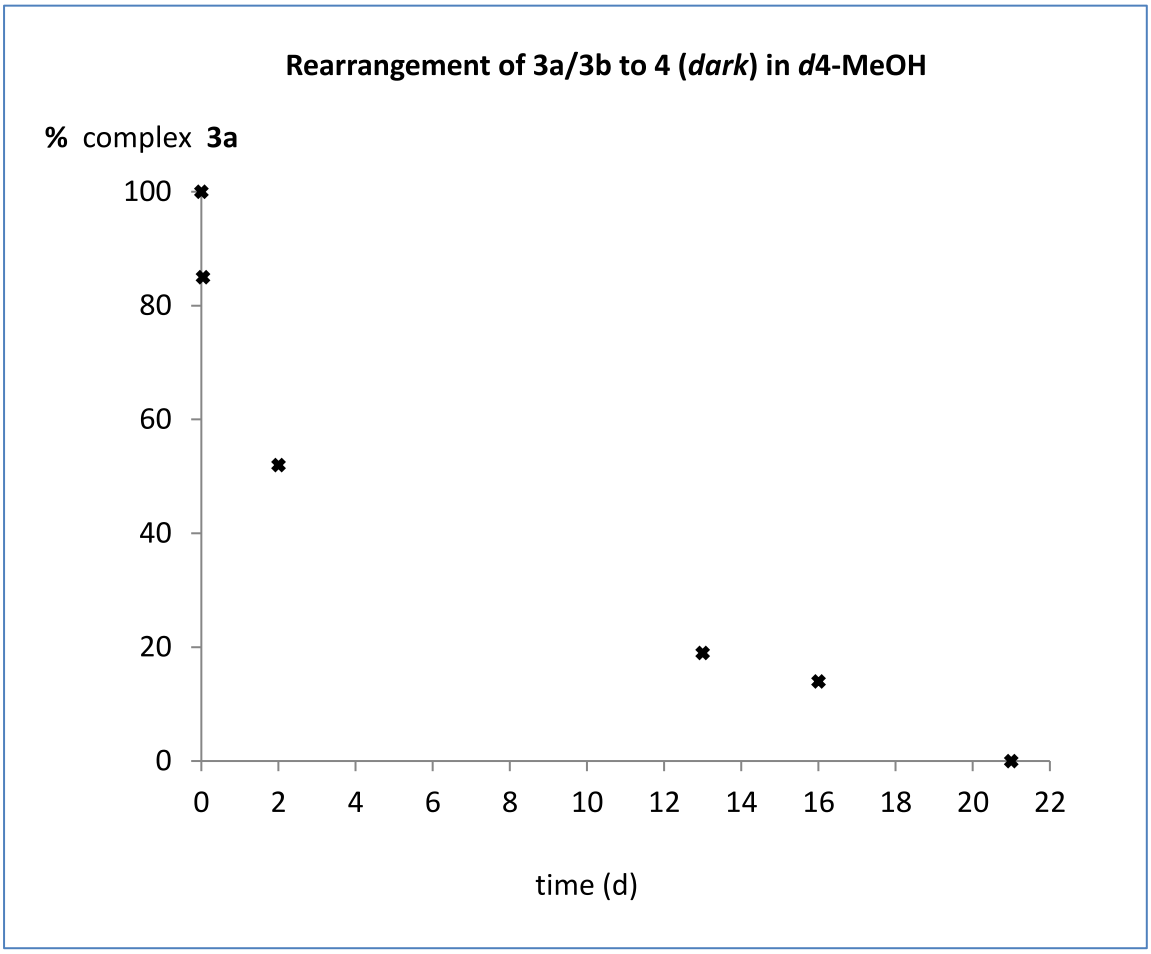

Figure S22. Conversion of **3a/3b** to **4** in the absence of light (d_4-MeOH) measured by normalised summed integration of Py_{ortho} ^{1}H NMR spectral resonances for **3a** (4H, at 8.93 ppm) and **4** (4H at 8.54 ppm).

ESI-MS of complex **4**

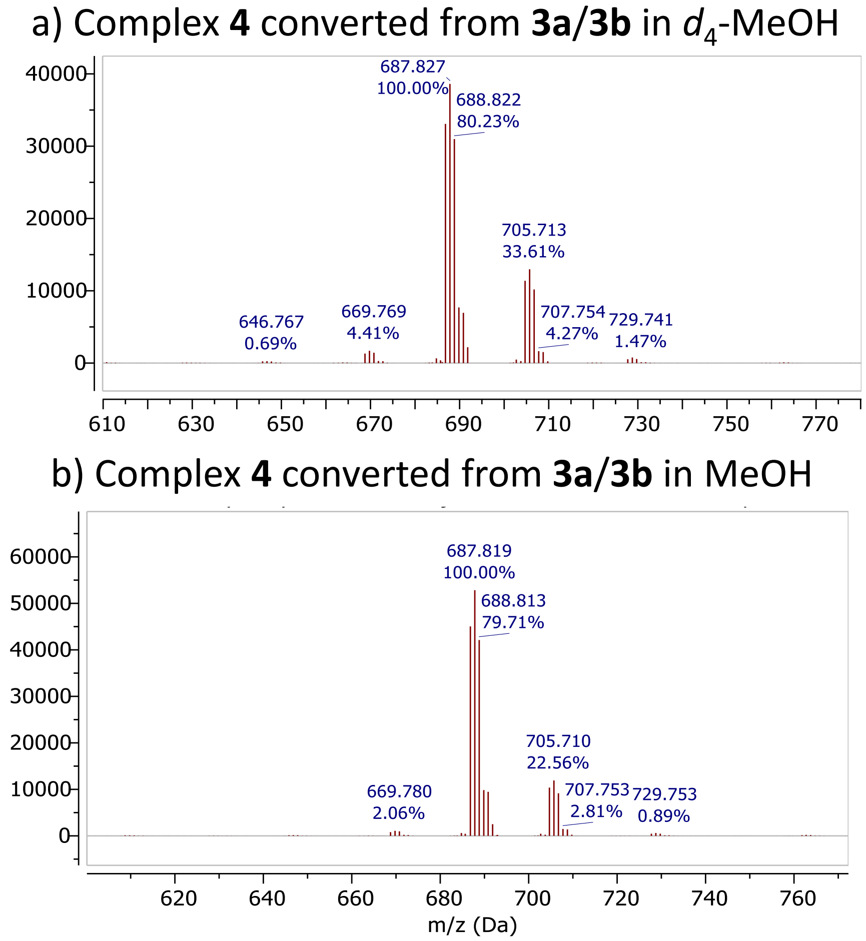

Figure S23. ESI-MS of complex **4** (1:1 H_2O/MeCN) which has fully converted from **3a/3b** in either a) MeOH or b) d_4-MeOH as reaction solvent.

^{1}H NMR spectroscopy of complex **4**

Complex **4** in MeOH-d_4

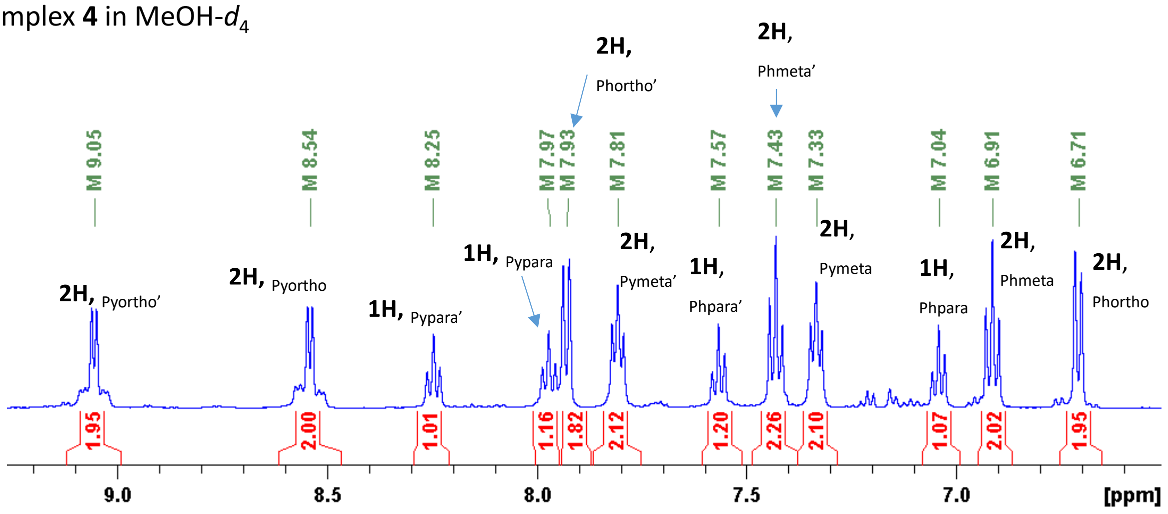

Figure S24. ^{1}H NMR (500 MHz) spectrum of complex **4** in d_4-MeOH.

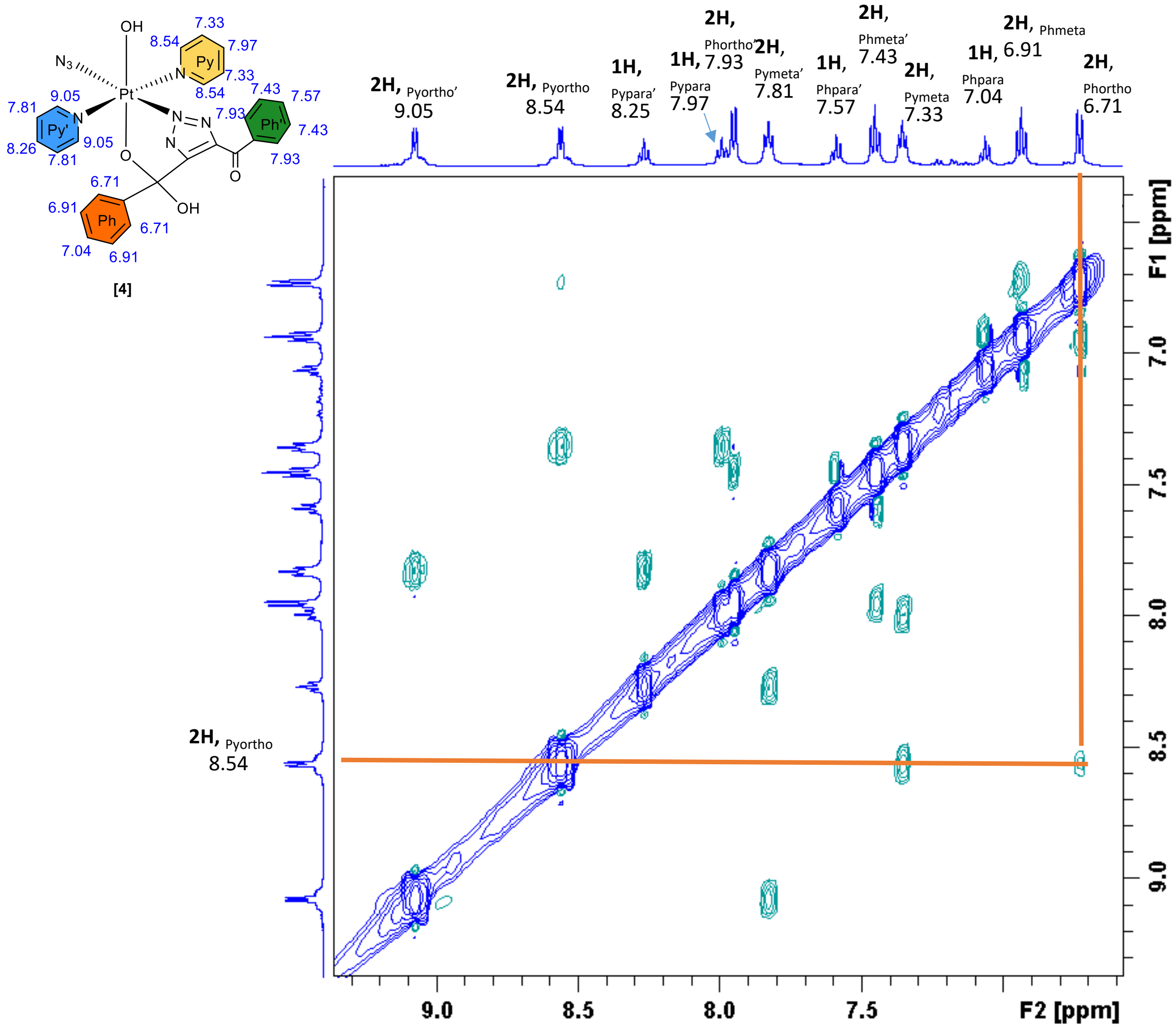

Figure S25. ^{1}H NMR NOESY (d_4-MeOH) of complex **4.**

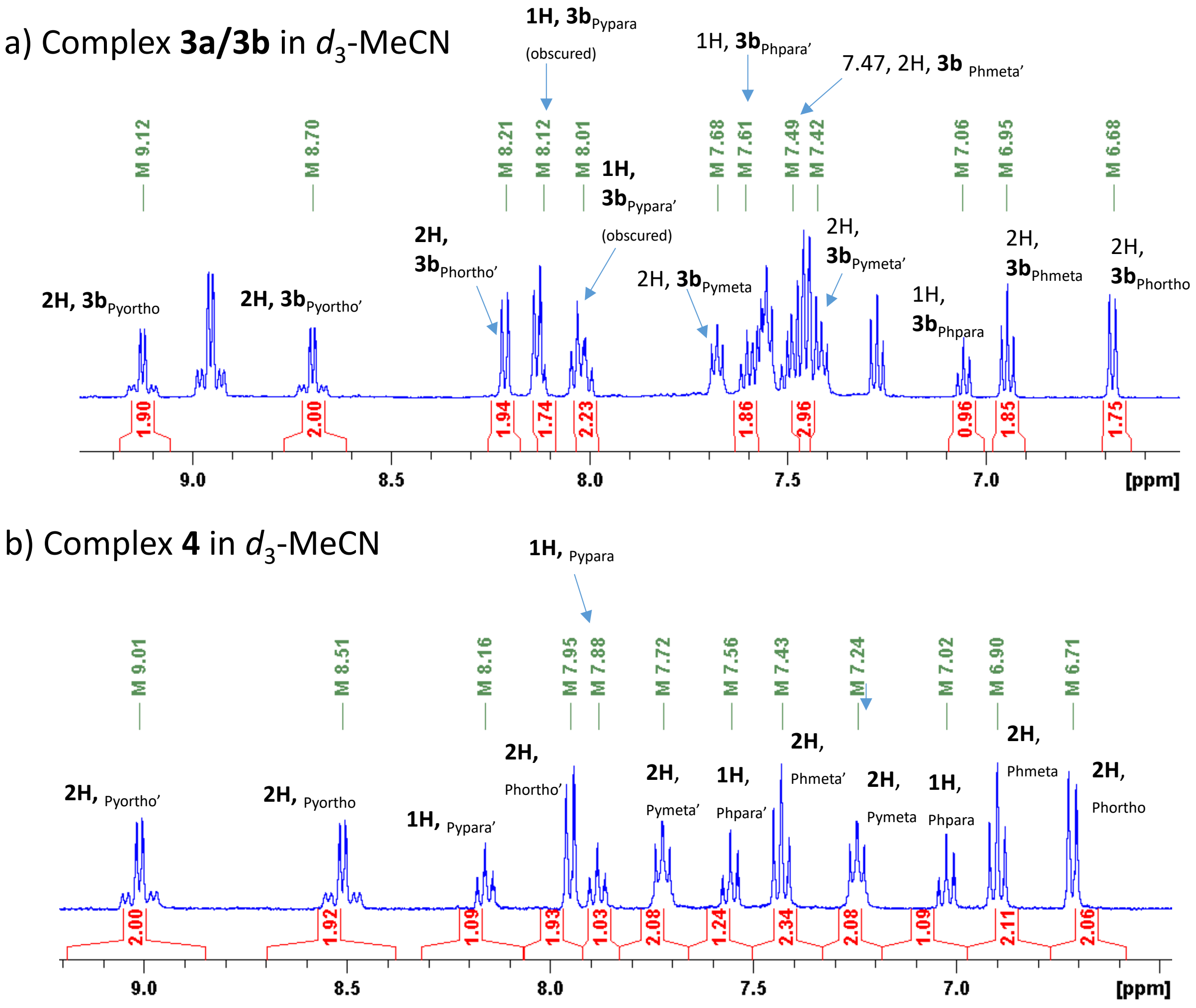

Figure S26. ^{1}H NMR comparison (d_3-MeCN) of **3a/3b** (only **3b** is labelled, for clarity) and **4** (converted from **3a/3b** in MeOH and reconstituted in d_3-MeCN).

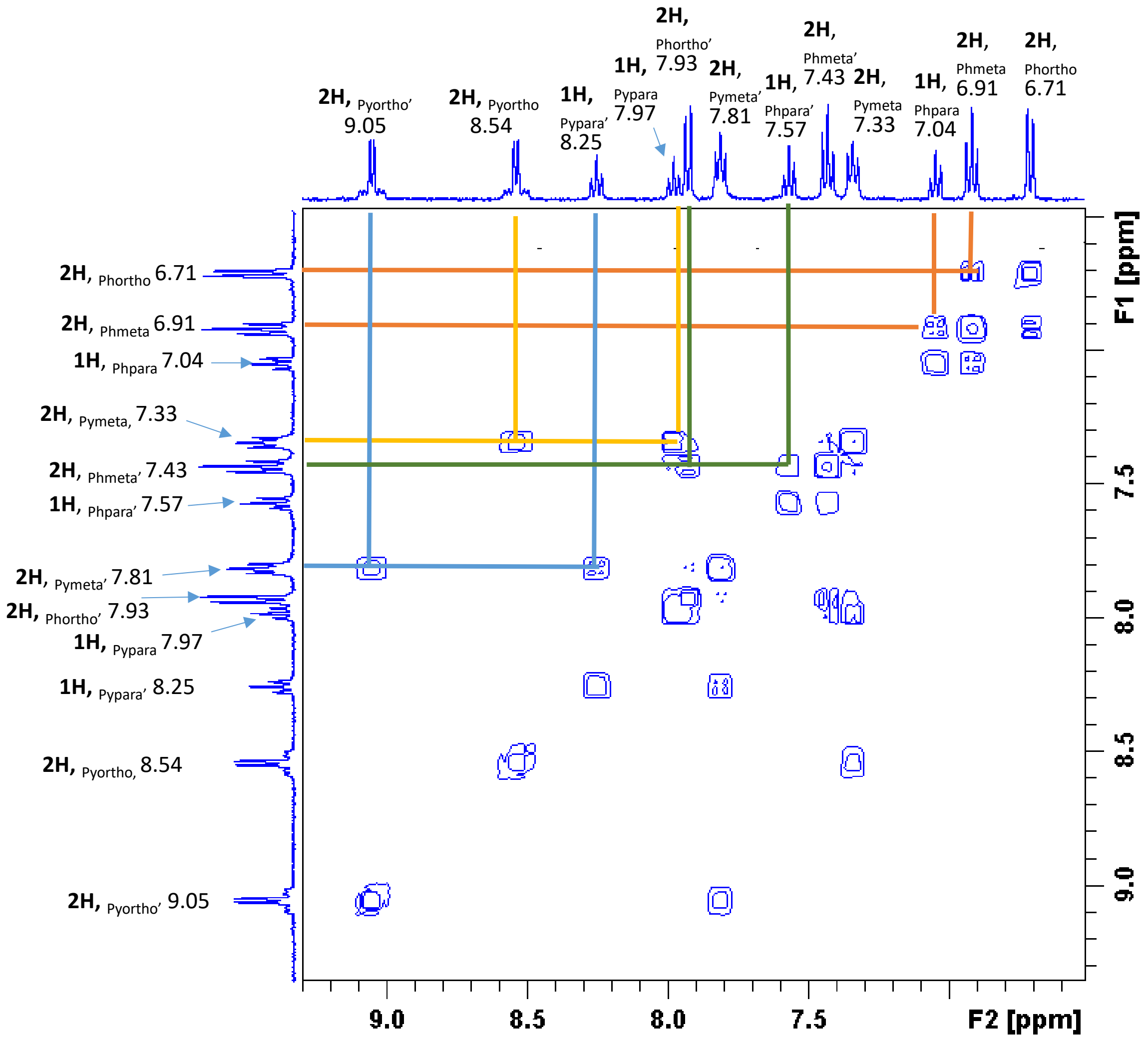

Figure S27. ^{1}H NMR COSY (400 MHz) spectrum of complex **4** in d_4-MeOH.

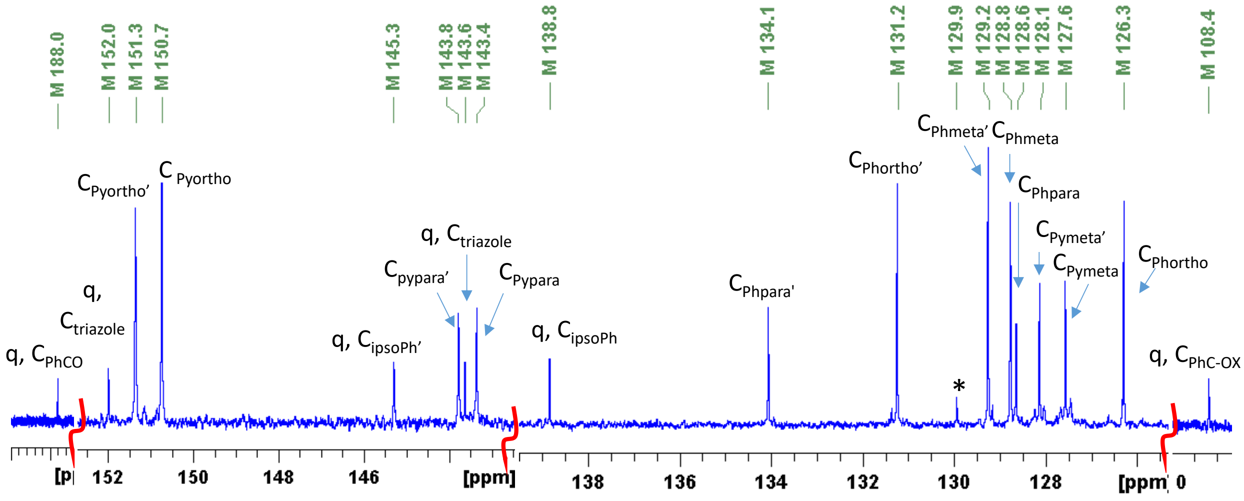

Figure S28. ^{13}C NMR (126 MHz) of complex **4** in d_4-MeOH.

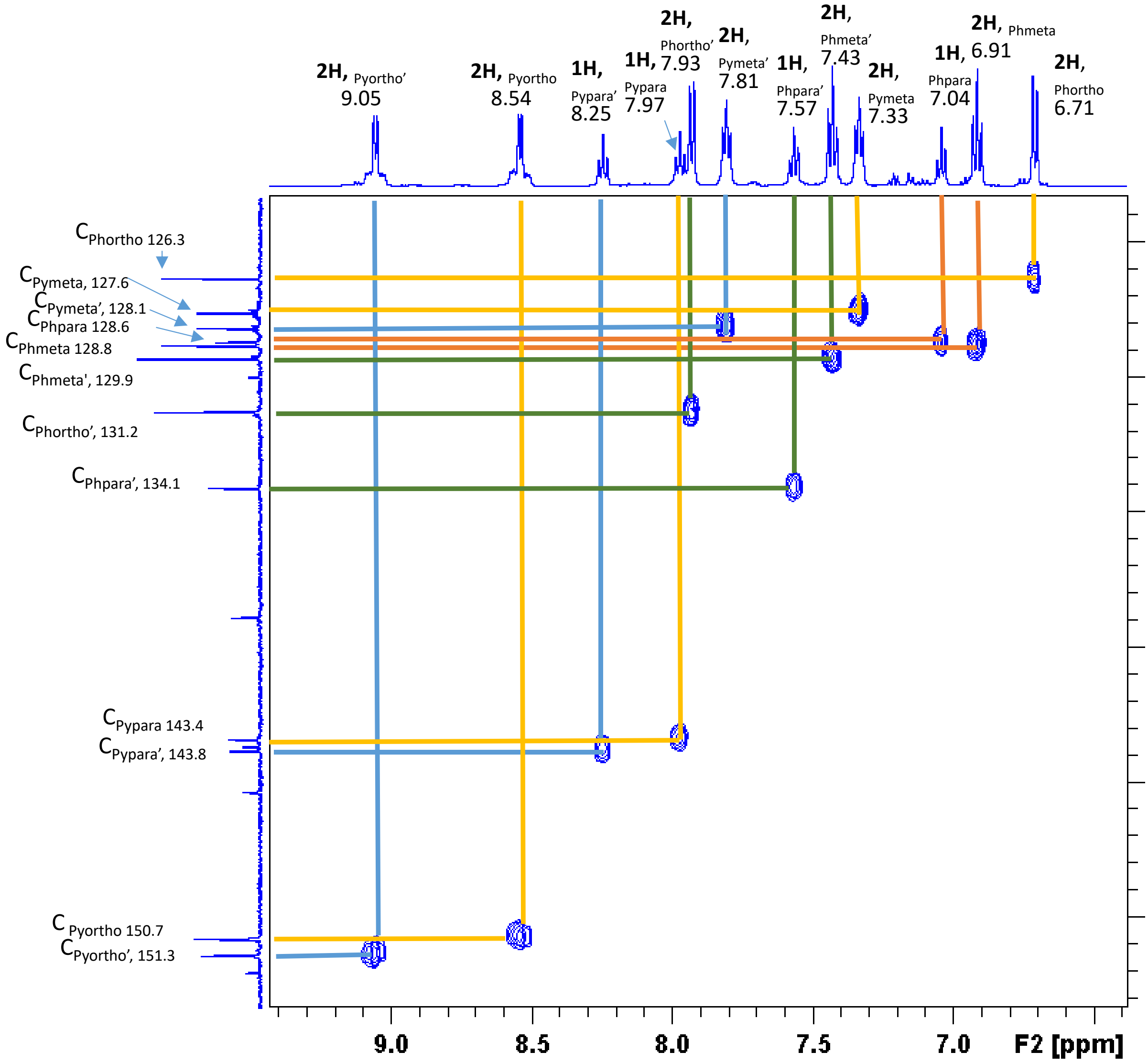

Figure S29. ^{13}C HSQC NMR (126 MHz) of complex **4** in *d*$_4$-MeOH.

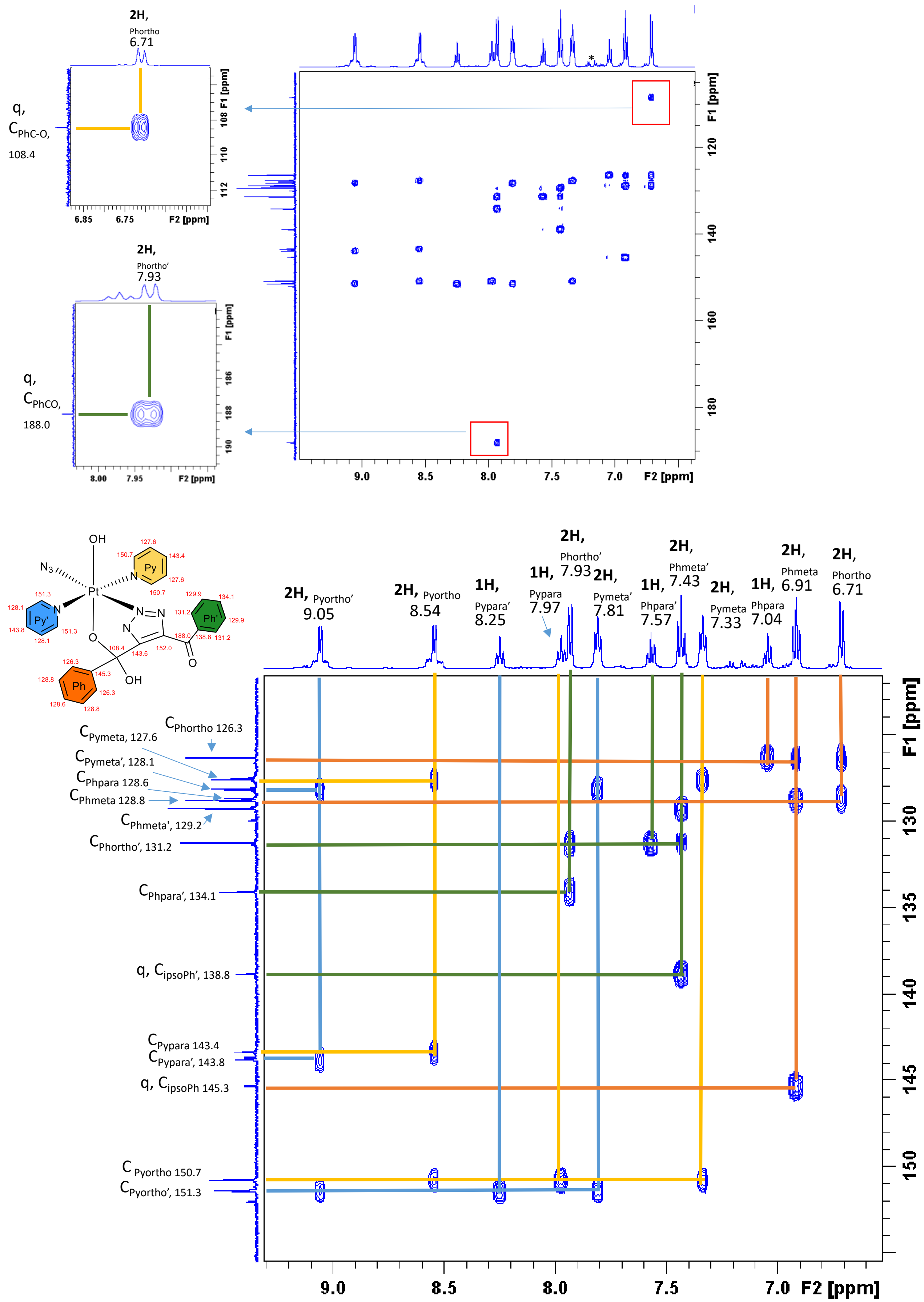

Figure S30. ^{13}C HMBC NMR (126 MHz) spectra of complex **4** in d_4-MeOH.

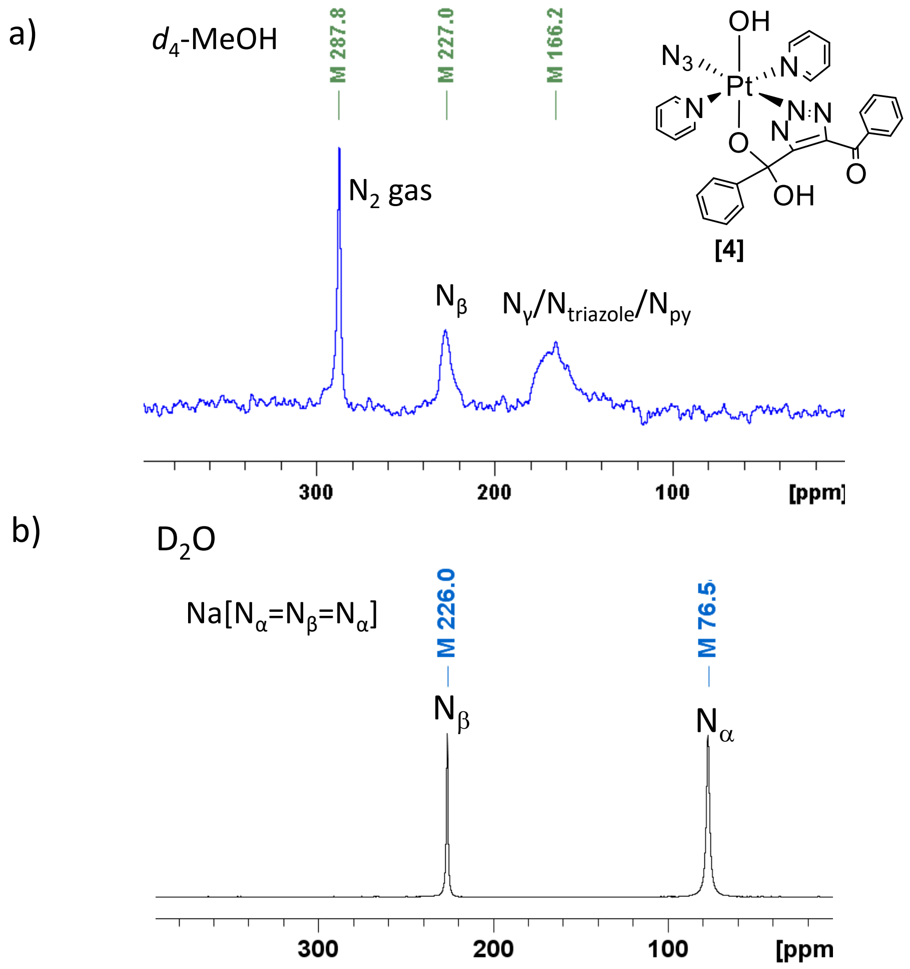

Figure S31. [14]N NMR spectra (36.1 MHz) of a) complex **4** in d_4-MeOH (8k data points, 358k scans, expt 23h48 b) NaN_3 in D_2O (0.3 M, ns 32k, 1.5h). (N.B. N_2 gas is observed more readily in d_4-MeOH than in D_2O, as previously reported).[3]

IR spectra

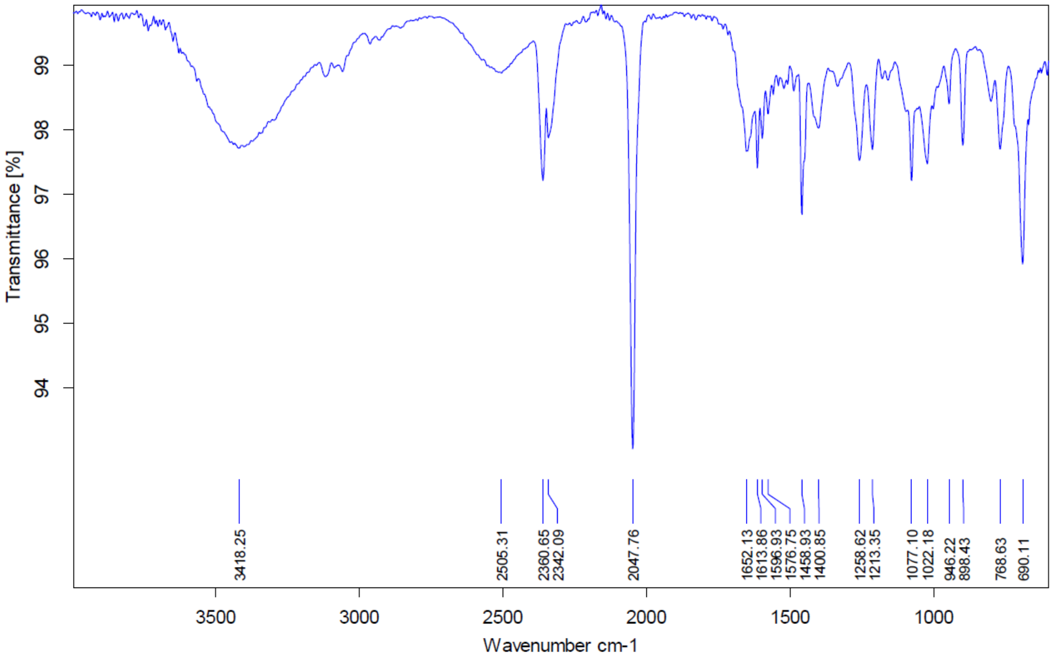

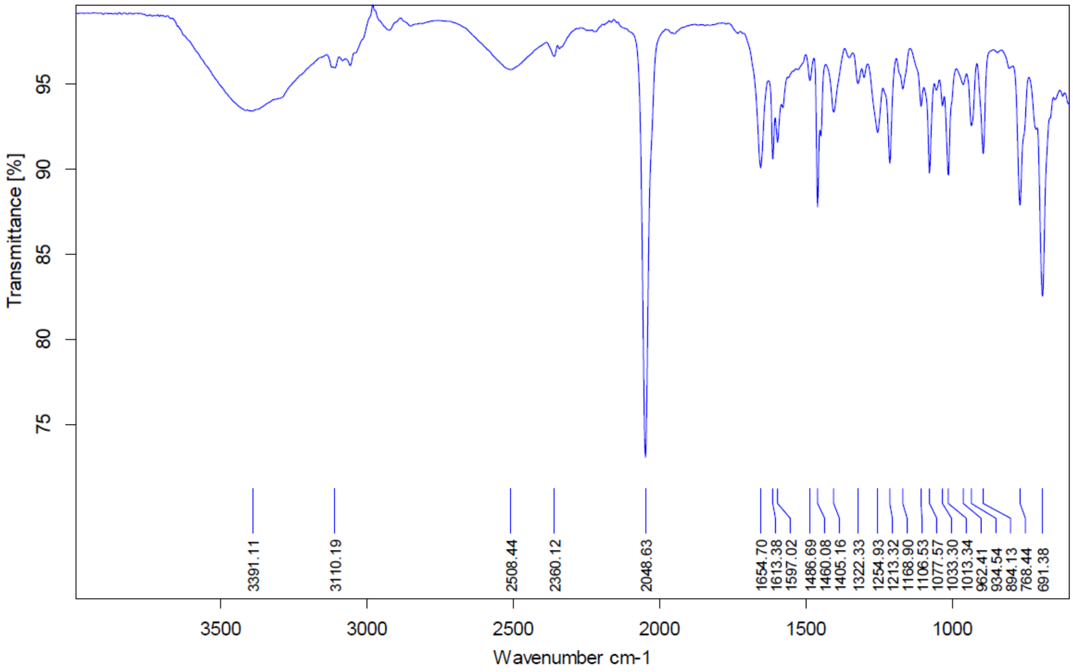

Figure S32. IR spectra of a) **3a**/**3b** before conversion to **4** (d_4-MeOH) and c) **4** after conversion (d_4-MeOH).

UV-Vis spectra of 4

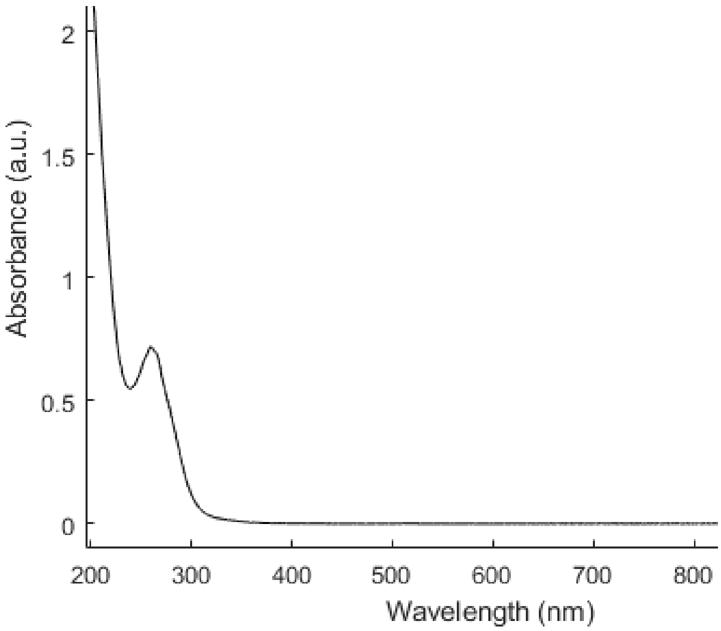

Figure S33. UV-vis spectrum of complex **4** (1:9 MeCN:H_2O).

Photochemistry

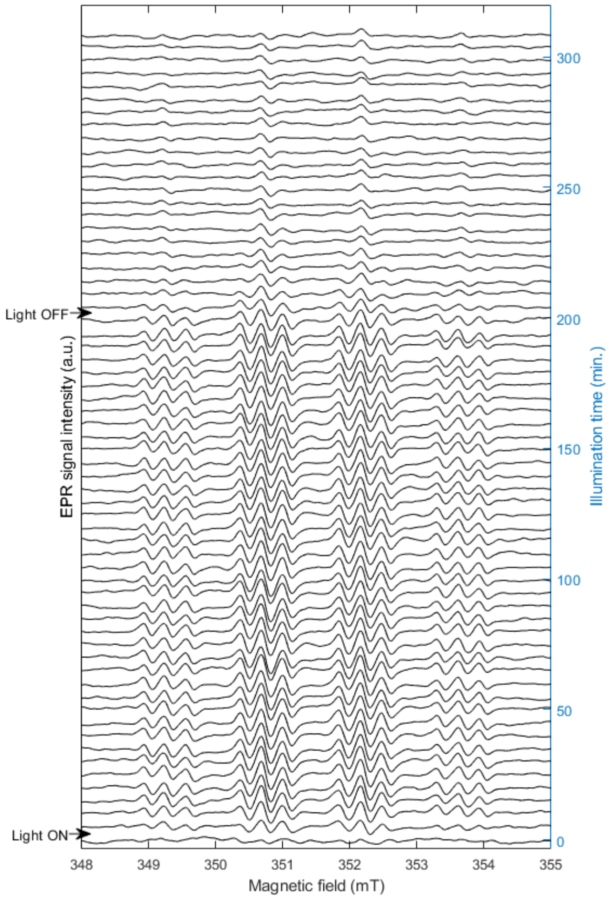

Figure S34. Cw-EPR spectra of the spin trapping experiment with **3a/3b** (10.5 mM Pt) and DMPO (21 mM) in H_2O under illumination (440 – 480 nm). Each spectrum was averaged for 5 min.

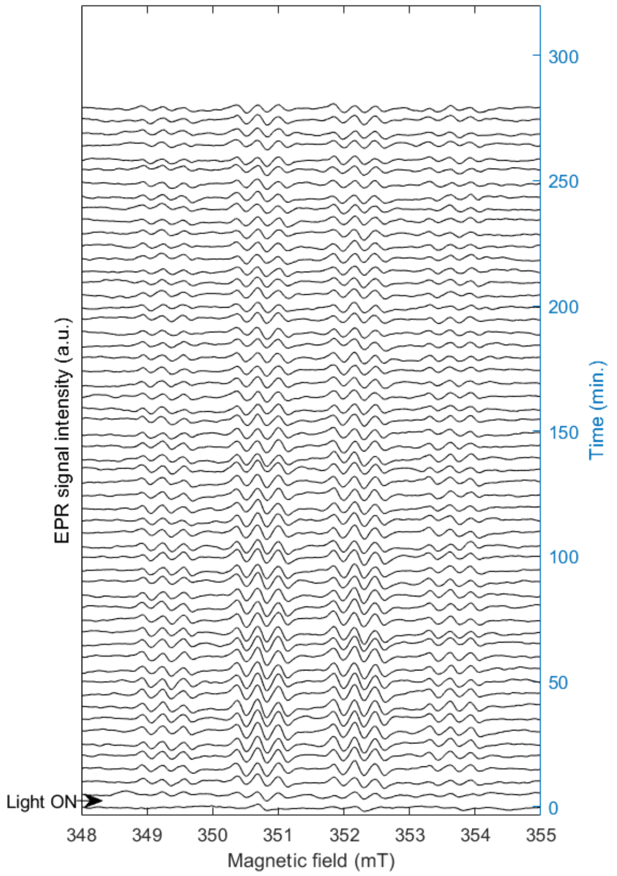

Figure S35. Cw-EPR spectra of the spin trapping experiment with **5**.H_2O_2 (< 1 mM Pt) and DMPO (21 mM) in H_2O under illumination (440 – 480 nm). Each spectrum was averaged for 5 min.

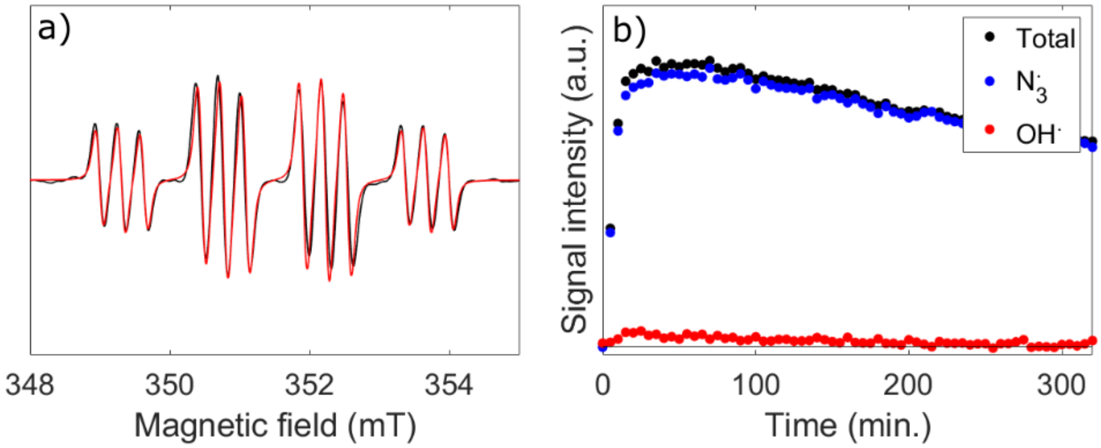

Figure S36. EPR spin trapping experiment in H_2O under illumination (440 – 480 nm) with compound **4** (3.5 mM) using 21 mM DMPO. (a) Experimental spectrum averaged for 100 min under continuous illumination (black) and simulation with a DMPO•-N3 : DMPO•-OH molar ratio of 96 : 4 (red). The total radical adduct concentration determined from spectral integration is (9.1 ± 0.9) µM. (b) EPR peak height transients of the total radical adduct signal (black), DMPO•-N3 signal (blue) and DMPO•-OH signal (red).

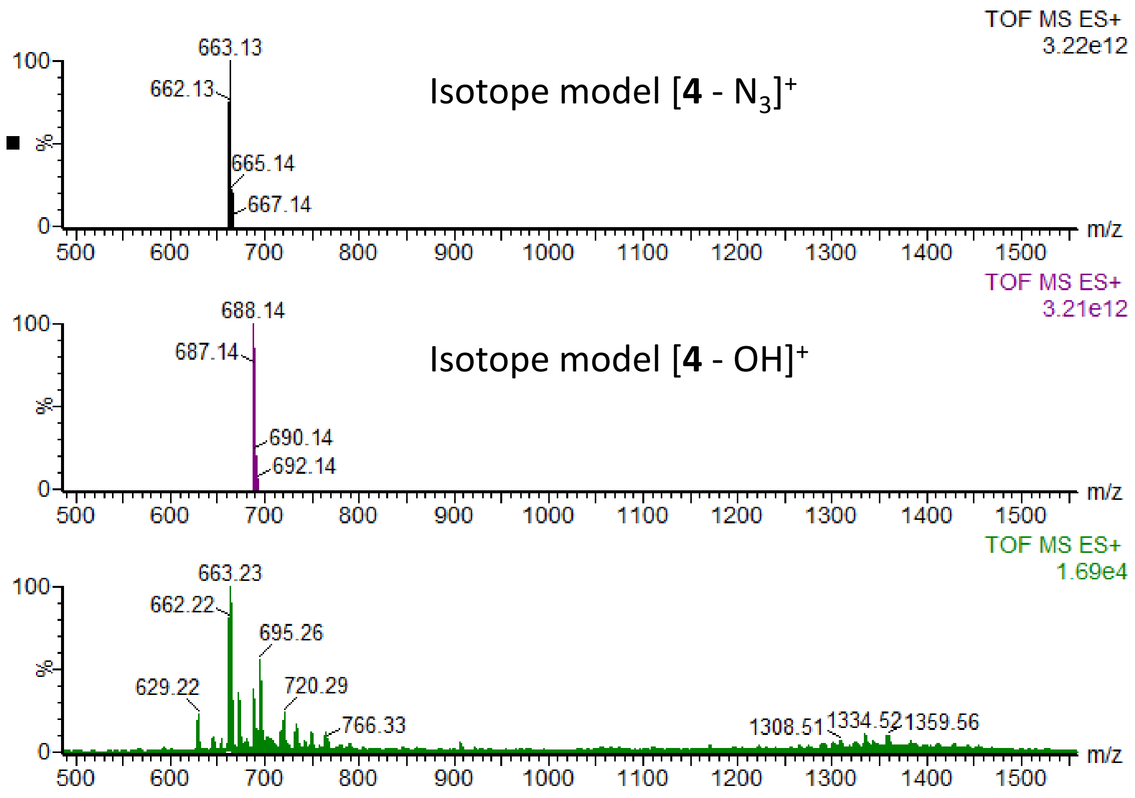

Figure S37. ESI-MS of solution of **4** in MeCN following 60 min irradiation with 452 nm light.

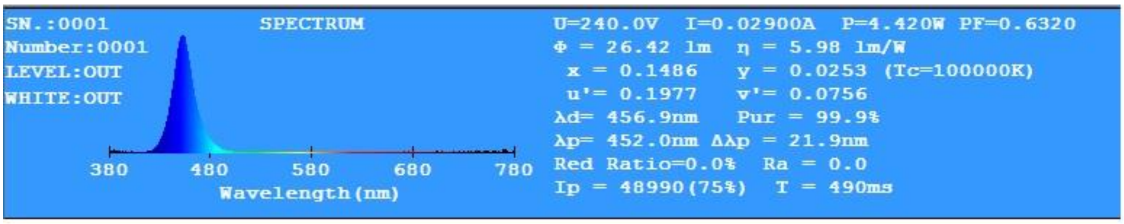

Figure S38. Spectral output of MiniSun (GU10 27 SMD) blue LED bulb, λ_{max} = 452 nm.

References

1 K. Yao, A. Bertran, J. Morgan, S. M. Hare, N. H. Rees, A. M. Kenwright, K. Edkins, A. M. Bowen and N. J. Farrer, *Dalton Trans.*, 2019, **48**, 6416.

2 O. V. Dolomanov, L. J. Bourhis, R. J. Gildea, J. A. K. Howard and H. Puschmann, *J. Appl. Crystallogr.*, 2009, **42**, 339.

3 N. J. Farrer, P. Gierth and P. J. Sadler, *Chem. - A Eur. J.*, 2011, **17**, 12059.
